# Chromate-Free Corrosion Protection Strategies for Magnesium Alloys—A Review: Part III—Corrosion Inhibitors and Combining Them with Other Protection Strategies

**DOI:** 10.3390/ma15238489

**Published:** 2022-11-28

**Authors:** Bahram Vaghefinazari, Ewa Wierzbicka, Peter Visser, Ralf Posner, Raúl Arrabal, Endzhe Matykina, Marta Mohedano, Carsten Blawert, Mikhail L. Zheludkevich, Sviatlana V. Lamaka

**Affiliations:** 1 Institute of Surface Science, Helmholtz-Zentrum Hereon, 21502 Geesthacht, Germany; 2 Departamento de Ingeniería Química y de Materiales, Facultad de Ciencias Químicas, Universidad Complutense de Madrid, 28040 Madrid, Spain; 3 Department of Functional Materials and Hydrogen Technology, Faculty of Advanced Technologies and Chemistry, Military University of Technology, 2 Kaliskiego Street, 00-908 Warsaw, Poland; 4 AkzoNobel, 2171 AJ Sassenheim, The Netherlands; 5 Henkel AG & Co. KGaA, 40589 Düsseldorf, Germany

**Keywords:** corrosion, inhibitor, magnesium, hexavalent chromium, self-healing

## Abstract

Owing to the unique active corrosion protection characteristic of hexavalent chromium-based systems, they have been projected to be highly effective solutions against the corrosion of many engineering metals. However, hexavalent chromium, rendered a highly toxic and carcinogenic substance, is being phased out of industrial applications. Thus, over the past few years, extensive and concerted efforts have been made to develop environmentally friendly alternative technologies with comparable or better corrosion protection performance to that of hexavalent chromium-based technologies. The introduction of corrosion inhibitors to a coating system on magnesium surface is a cost-effective approach not only for improving the overall corrosion protection performance, but also for imparting active inhibition during the service life of the magnesium part. Therefore, in an attempt to resemble the unique active corrosion protection characteristic of the hexavalent chromium-based systems, the incorporation of inhibitors to barrier coatings on magnesium alloys has been extensively investigated. In Part III of the Review, several types of corrosion inhibitors for magnesium and its alloys are reviewed. A discussion of the state-of-the-art inhibitor systems, such as iron-binding inhibitors and inhibitor mixtures, is presented, and perspective directions of research are outlined, including *in silico* or computational screening of corrosion inhibitors. Finally, the combination of corrosion inhibitors with other corrosion protection strategies is reviewed. Several reported highly protective coatings with active inhibition capabilities stemming from the on-demand activation of incorporated inhibitors can be considered a promising replacement for hexavalent chromium-based technologies, as long as their deployment is adequately addressed.

## 1. Foreword

Magnesium (Mg) and its alloys are characterized by their low density and high specific strength, which make them competitive alternatives to traditional structural materials in aerospace and automotive industries. However, Mg alloys are prone to corrosion, particularly when in contact with aqueous solutions. While this property of magnesium alloys is taken as an advantage in the field of biodegradable metallic implants and primary Mg–air batteries, it stifles magnesium utilization for structural applications in aerospace and automotive. The traditional approach to suppress the corrosion of magnesium and other metals is to apply a coating system, which acts as a barrier against the penetration of corrosive species toward the metal surface. In a complementary approach, corrosion inhibitors are added to a corrosive medium or a protective coating to further retard the corrosion of the exposed metallic part [1]. Inhibitors are widely used for various industrial applications, including the protection of engineering parts, pipelines, and petroleum-handling facilities [2]. Application of inhibitors is considered a cost-effective, easy-to-operate, and practical method for corrosion control. The best-known chemical with inhibitive features is hexavalent chromium, which functions as a panacea to the corrosion susceptibility of many engineering metals. However, due to the strict rules and regulations imposed by environmental agencies, the utilization of hexavalent chromate has been limited [3]. In particular, in Europe, the use of Cr(VI) substances is regulated by the European Chemical Agency, an agency of the European Union that composed an authorization list, also known as the list of substances included in Annex XIV of REACH [4]. Three leading chromate-containing substances have been banned since 21 May 2015. The majority of the other Cr(VI) compounds have been banned since 21 September 2017, while strontium chromate and three more Cr(VI) compounds were banned starting from 22 January 2019 [5]. Since no adequate substitutes for chromates have been adapted in a timely manner by concerned industries, a number of key players in the European space industry have initiated the Space Chromate Task Force, aiming to postpone the ban to use Cr(VI) compounds in aerospace industry [6]. As a result, the sunset date for the use of Cr(VI) compounds for surface treatment for a number of companies related to aeronautics and aerospace industries has been postponed until the year 2024 [7]. Nevertheless, the use of Cr(VI) is expected to be prohibited for all industries in the future. In light of these factors, the search for new corrosion inhibitors suitable for industrial applications is urgent and extremely pertinent. Thus, numerous recent scientific works have been dedicated to the discovery of adequate substitutes for chromates.

**Figure 1:**
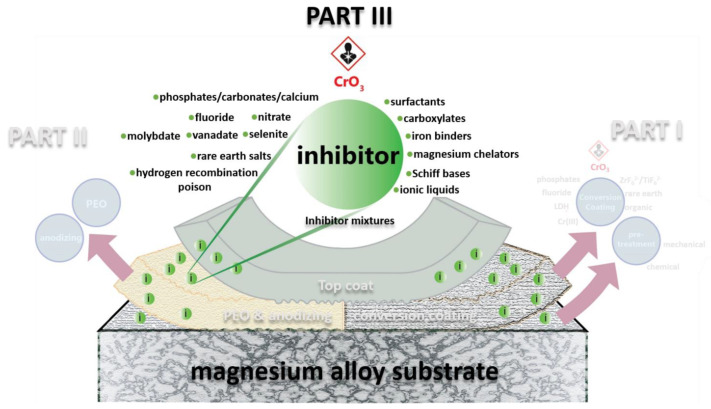
Schematic of review trilogy. This work, as **PART III** of the review, has been highlighted with more vivid colors, in contrast to the dimmed color of **PART I** and **PART II**.

This review is the third part of a trilogy on Cr(VI)-free corrosion protection strategies for magnesium alloys. In **PART I** of the review [8], conversion coating technologies are reviewed as one of the most widely used methods of protecting metal surfaces. As a controlling step for the performance of a conversion coating, pre-treatment of magnesium surface is also discussed in **PART I**. **PART II** of the review [9] focuses on Plasma Electrolytic Oxidation (PEO) coating as one of the highly developed methods to protect magnesium surface in the recent years. This part of the review (**PART III**) focuses on corrosion inhibitors for magnesium and approaches to incorporate them into coating systems. An overview of the review trilogy is illustrated in the graphical abstract of all three review parts. Figure 1 high-lights the outline of **PART III** with the most promising corrosion inhibitors and inhibitor classes.

In contrast with coatings, which aim to isolate the whole surface of an engineering part from corrosive media, inhibitors are able to target specific regions of the metal surface (the cathodic region and the anodic region). In this context, inhibitors can be classified as **cathodic**, **anodic**, or **mixed** types. Another way to categorize inhibitors is based on their interaction with a metal surface in corrosive media. Inhibitors can precipitate in the form of an insoluble compound or can be adsorbed on the surface of metal: **precipitation**, **passivation**, or **adsorption** inhibitors. The distinction between these three groups is subtle, especially with the passivation and precipitation types. The latter is deposited as a partially protective layer (e.g., cerium and lanthanum hydroxides), while the former is similar to conversion coating, forming a layer of sparingly soluble magnesium products (phosphates and fluorides). Many of the inhibitors that form precipitation layers on the surface can also be considered as conversion coating precursors (see **PART I** of this review [8]). For instance, chromate has been frequently addressed in the literature as either an inhibitor or a chemical used to form conversion coatings. Although they follow a similar protection mechanism, technical features can distinguish them as belonging to distinct categories. The main difference is that inhibitors are present in the corrosive medium throughout the service life of an engineering component. In contrast, precursors for conversion coatings are removed from the media when the coating formation is finished. Moreover, conversion coating solution baths are usually at low pH levels and contain oxidizing agents in order to accelerate the dissolution of the magnesium substrate. On the contrary, inhibitors are typically incorporated into coating systems under conditions that are the least corrosive to the coating system.

There are numerous factors affecting the chemistry of the interaction between inhibitor and metal. Small variations in each factor may result in very different inhibition behaviors. This must always be kept in mind when different scientific work results are being compared; for instance, in the case of magnesium alloys, a minor variation in Al and Zn content in alloy composition can activate or deactivate the inhibitive properties of many inhibitors [10].

This review focuses neither on magnesium corrosion mechanisms nor on the methods used to measure corrosion rates. These aspects were extensively reviewed by Esmaily et al. [11]. The experimental methods for calculating the inhibiting efficiency (IE) are typically thoroughly discussed in the original papers and refer to the well-known relation, IE(%) = 100% × (ExpPar_blank_ − ExpPar_inh_)/ExpPar_blank_. ExpPar refers to measured experimental parameter; *blank* and *inh* refer to a blank testing medium, e.g., NaCl solution and that with added corrosion inhibitor, respectively. Most typical measured experimental parameters include weight loss, volume of evolved hydrogen, polarization resistance, or low-frequency impedance accessed from EIS measurements (Electrochemical Impedance Spectroscopy), and corrosion current density calculated from potentiodynamic polarization curves.

In this review, the substances used as inhibitors for magnesium alloys are first categorized as organic and inorganic inhibitors, since this leads to a comparable interaction between inhibitors and metal surface in each category. The corrosion inhibition mechanisms stand at the center of this review when placing inhibitors in a particular group. In each category, in addition to reviewing the important inhibitors that have been studied at length, the most promising and most recent inhibitors are discussed. Emerging approaches of inhibitor discovery using *in silico*, or computational, screening are briefly described.

## 2. Inorganic Inhibitors

As mentioned in the introduction, corrosion inhibitors with the ability to form precipitating compounds can also be classified as conversion coating precursors. This overlap between two categories mostly happens for inorganic substances such as chromate, vanadate, phosphate, and rare earth elements salts. Therefore, here, we briefly provide a review of some inorganic inhibitors studied for magnesium alloys, while the complementary information about conversion coatings formed based on these inhibitors has been provided in **PART I** of the review [8].

### 2.1. Chromate Based Inhibition

Hexavalent chromium is a panacea for corrosion of engineering metals. Compounds containing hexavalent chromium remain among the most effective inhibitors, with superior protective properties for steel, aluminum alloys, and magnesium alloys [12]. 

Chromate or hexavalent chromium (Cr(VI)) is a strong oxidizing agent that is reduced to trivalent chromium by oxidizing the magnesium on the surface. Then, the hydroxide Cr(OH)_3_ and oxide Cr_2_O_3_ of chromium co-precipitate on the Mg surface to form a mixed hydroxide compound [13,14]. Apart from Cr(III), this complex film contains trapped residual hexavalent chromium that acts as the active inhibitor and provides the ability of film “regeneration” [15,16]. Trapped hexavalent chromium is enriched more in the outermost layer of the film, since reducing Cr(VI) by magnesium is less probable due to coverage of the surface [17]. However, the chromates trapped in the formed film have relatively high mobility, and are thus capable of reaching the defects and locally re-passivating the exposed area [18].

Although the excellent corrosion inhibition effect of chromate-based inhibitors has been known for decades, the exact mechanism of inhibition is still not fully understood, especially for Mg alloys. Williams et al. [19] reported a mechanism for cathodic site depolarization by chromate ions based on SVET measurement over AZ31 magnesium alloy from the initial stages of immersion in an aerated 5% *w/v* aqueous NaCl solution containing Na_2_CrO_4_ salt. They observed very intense cathodic “hotspots” over the exposed surface, which decreased in number per unit area over time of immersion. Moreover, these cathodic hotspots have a limited lifetime (less than 15 min), emerging and disappearing at different locations over the scan area. Based on the SVET observations, they concluded that this transient intense local cathodic current is due to the reduction of Cr(VI) oxyanions to Cr(III) and hydrolysis of the Cr(III) species according to the following reaction, rather than typical cathodic hydrogen evolution reaction:

(1) $$CrO_{4}^{2-}\left(aq\right)+4H_{2}O+3e^{-}⇆Cr{\left(OH\right)}_{3}\left(s\right)+5OH^{-}$$

The abovementioned reaction occurs mostly on cathodic sites where the elevated local pH imposes more precipitation of chromium hydroxide, which prevents further cathodic activity. 

### 2.2. Phosphates 

Phosphates have been extensively researched as an alternative to chromate-based inhibitors due to their environmentally friendly properties and comparatively high protective properties for magnesium alloys. Numerous phosphate solutions modified with different organic and inorganic additives have been reported, mainly with the aim of achieving a conversion coating on magnesium alloys with higher protective behavior (see **PART I** of this review [8]). However, only a few studies have addressed the mechanism of corrosion inhibition caused by phosphate ions in corrosive media. 

Depending on the pH of the solution, phosphate ions can be protonated to different degrees. Tribasic phosphate anions PO_4_^3−^, which predominantly exist at elevated pH, can bond with Mg^2+^ ions and form sparingly soluble Mg_3_(PO_4_)_2_ (see Reaction (2)) that is believed to play the main role in the inhibition of magnesium alloys [16]. Other compounds of magnesium with phosphates such as Mg(H_2_PO_4_)_2_ and MgHPO_4_ are relatively soluble in aqueous environments and do not contribute to magnesium surface passivation [20].

(2) $$3Mg^{2+}\left(aq\right)+2PO_{4}^{3-}\left(aq\right)⇆\;Mg_{3}{\left(PO_{4}\right)}_{2\;}↓$$

**Figure 2:**
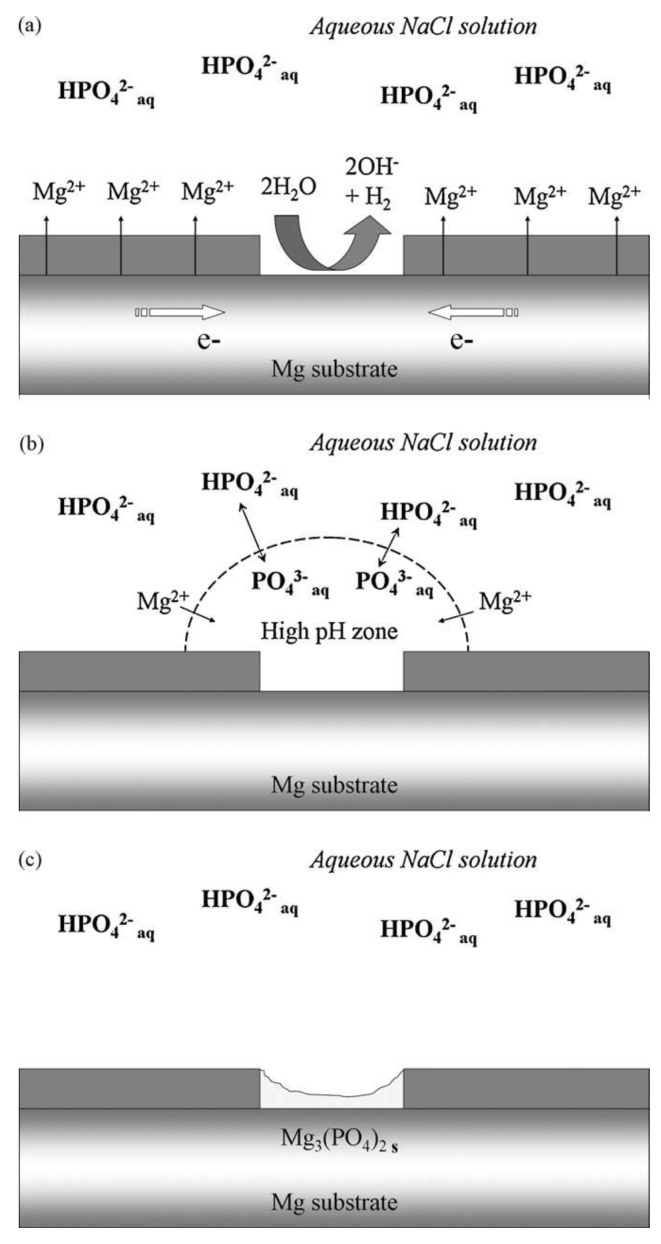
Schematic representation of the mechanism of magnesium corrosion inhibition by aqueous phosphate ions at neutral pH, showing (**a**) localization of the early stages of corrosion, (**b**) phosphate speciation in the vicinity of the local cathode, and (**c**) deposition of an insoluble film [21]. Adapted from [21] with permission from Elsevier.

pH increase in the vicinity of cathodic sites such as iron-rich impurities and second phases results in the deprotonation of HPO_4_^2−^ species to PO_4_^3−^. Free magnesium ions produced by oxidation of magnesium surface migrate to the regions with a high concentration of PO_4_^3−^ and form solid Mg_3_(PO_4_)_2_ [21]. Figure 2 depicts a schematic representation of corrosion inhibition by phosphate anions at neutral or slightly acidic pH (between 4–7), with HPO_4_^2−^ as the dominant specie. At higher pH values, the inhibition efficiency is reduced due to the precipitation of Mg_3_(PO_4_)_2_ away from the Mg surface. 

Therefore, Mg_3_(PO_4_)_2_ is more likely to precipitate on cathodic sites, on which local alkalization occurs due to the cathodic reactions. Consequently, phosphates are usually considered cathodic inhibitors and shift E_corr_ to more negative values [19]. Preferential precipitation of Mg_3_(PO_4_)_2_ on the beta phase in Mg-Li alloy [22] and AZ91D alloy [23] is also in agreement with the abovementioned mechanism. On the other hand, it has been shown that local pH on Mg surface reaches a value of above 10 within the first minute of exposure to the electrolyte [24]. Hence, phosphate precipitation should normally occur homogeneously on the entire surface. 

Diammonium phosphate (NH_4_)_2_HPO_4_ is also reported to provide a high inhibiting effect on magnesium alloys with co-precipitation of Mg_3_(PO_4_)_2_ and MgNH_4_PO_4_ [25,26]. Relatively high dissolution of AM60 alloy has been observed instantaneously in (NH_4_)_2_HPO_4_ solution that is substantially reduced within a few h due to the formation of a denser protective film.

### 2.3. Rare Earth Salts

Rare earth salts, especially cerium salts, have also been intensively used as a possible candidate for the replacement of chromate-base inhibitors to protect non-ferrous alloys. Their exact inhibition mechanism on magnesium alloys is still under critical scrutiny. Sparingly soluble cerium hydroxides are believed to be the main components of the protective layer on the magnesium substrate when it is immersed in a cerium-containing solution. 

Montemor et al. [27] observed that the film formed on AZ31 Mg alloy at the very early stage of immersion (less than 1 min) in 1.0 × 10^−3^ M Ce(NO_3_)_3_ is mainly composed of Mg oxide/hydroxide. The cerium content in the formed film is enriched after 1 min of immersion, and for a longer immersion time, the concentration of Ce becomes approximately constant, mainly reflecting film thickening.

Williams et al. [19] observed a poor corrosion inhibition on AZ31 alloy by addition of 10^−2^ M Ce(NO_3_)_3_ into the 5% *v/w* NaCl solution. The critical pH value for precipitation of cerium (III) hydroxide was obtained as pH 8.5, which is a near-neutral pH value. Therefore, they suggested that cerium (III) hydroxide precipitation occurs in the bulk electrolyte above the corroding surface, leading to inefficient protection properties. Similar results were observed, and the same conclusions were drawn by Lamaka et al. [10] with regard to Ce^3+^ and La^3+^ salts. It is crucial that when inhibitors are added to the aqueous solution as salts, the effect of both anions and cations must be taken into consideration. For instance, in the case of Ce-base inhibitors, Ce(NO_3_)_3_ salt exhibits good corrosion resistance for Mg alloys, whereas CeCl_3_ may even accelerate the corrosion rate [10,19]. This can be related to the increase in corrosiveness of the electrolyte when additional chlorides are introduced. More importantly, there is clear evidence [10,28] of the strong inhibition effect of NO^−^_3_ on magnesium alloys that can synergistically boost the overall inhibition effect when it is present along with Ce^3+^ ions in the electrolyte. 

It is worth mentioning here that, despite a few reports on the remarkable inhibition effects of NO_3_^−^ on the corrosion of magnesium and its alloys [10,28,29,30], the inhibition mechanism has not been addressed in detail.

### 2.4. Inhibition Preventing Hydrogen Re-Combination 

**Figure 3:**
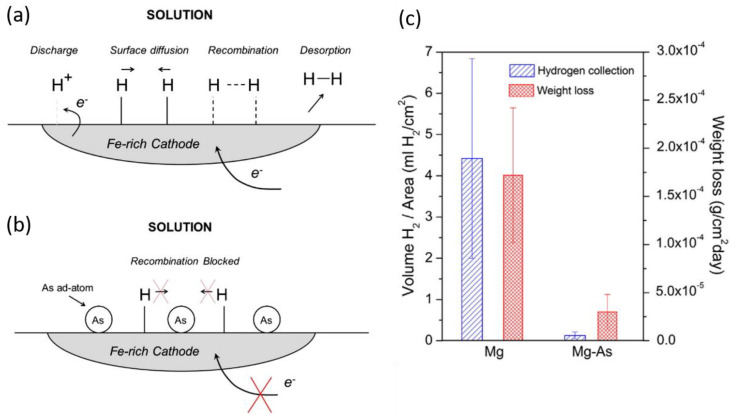
(**a**,**b**) Schematic representation of the mechanism of hydrogen recombination poisoning and (**c**) decreased corrosion rate in presence of arsenic alloyed with Mg [32]. Adapted from [32] with permission from Elsevier.

Another class of corrosion inhibitors has been introduced by Williams, McMurray, and Birbilis [31,32,33]. Arsenic species, either soluble arsenates added to corrosive medium [31] or metallic arsenic alloyed with magnesium [32,34], exhibited marked cathodic inhibition owing to the ability of arsenate to inhibit hydrogen atom recombination, and thus the cathodic H_2_ evolution, Figure 3a–c. The poisoning mechanism is based on the reduction of arsenates in the electrolyte to metallic arsenic on Fe-rich impurities in the Mg matrix. At elevated pH values, analogous to phosphates, the precipitation of Mg_3_(AsO_4_)_2_ in the electrolyte away from the Mg surface leads to the depletion of arsenates close to the substrate. Thus, the poisoning of the HER is reported to be more effective in the acidic condition below four. Furthermore, the precipitation of insoluble Mg_3_(AsO_4_)_2_ (K_sp_ = 2 × 10^−20^ at 25 °C) itself plays a role in the corrosion inhibition of magnesium alloys. Considering the toxicity of arsenic, there is a small likelihood that any water-soluble arsenic species can find an unrestricted commercial application as a corrosion inhibitor. 

A similar advantageous poisoning effect has also been reported via alloying of magnesium with germanium [33,35], antimony [36], and indium [37,38]. Thus, a similar effect is expected from the addition of their soluble salts to the electrolyte, rendering them potential corrosion inhibitors. In addition to the advantageous poisoning effect of indium on the water reduction cathodic reaction, precipitation of In(OH)_2_ on the surface can contribute to the corrosion inhibition of Mg substrate [39]. However, in common with the adverse effect of Ce^3+^ and La^3+^ ions in the electrolyte, the formation of In(OH)_2_ can hinder the increase in the local pH close to the Mg substrate, which may lead to a higher corrosion rate [37,39].

In addition to the reduction of metals and their salts on Fe-rich impurities, a recent report by Mercier et al. [40] have reported adsorption of sulfur on Fe-rich impurities from H_2_S_aq_ in the electrolyte. As a result, a significant reduction in HER at OCP and anodic polarization (OCP + 0.25 V and OCP + 0.5 V) was observed. 

### 2.5. Other Inorganic Inhibitors

There are several inorganic inhibitor systems reported to effectively reduce the corrosion rate of Mg alloys, but limited studies have been conducted on them. Many of the related studies are promising in terms of performance and environmental impact and are worthy of more investigation. Below, some of them are briefly reviewed:

**Fluorides**

Hydrofluoric acid (HF) has been intensively investigated and used as a component of pretreatment and conversion coatings on Mg, establishing a uniform passive thin layer made of partially hydrated magnesium fluoride MgF_2−x_OH_x_·yH_2_O or a mixture of Mg(OH)_2_ and MgF_2_ (see **PART I** of the review [8]). As an inhibitor in neutral or alkaline conditions, F^−^ has been shown to exhibit some degrees of inhibition effect on different Mg alloys [10,25,41,42,43]. Similar to the conversion coating based on HF, the formation of MgF_2_ film along with Mg(OH)_2_ on the Mg surface has been mentioned as the main inhibition mechanism. However, perovskite crystals (NaMgF_3_) are also frequently observed in such cases, which is promoted by the increase in F^−^ concentration [44,45]. Hydrofluoric acid also passivates Mg surface and was used to dissolve Ti phase from Mg-Ti micro-composite [46] or Ti-Ta-Cu phase from bicontinuous nanocomposite [47], leaving micro-porous or nanoporous magnesium, correspondingly.

**Bi/Carbonate**

Dissolved carbonate anions (HCO_3_^2−^ and CO_3_^−^) are able to reduce the corrosion rate of magnesium alloys through the formation of insoluble magnesium carbonate [48]. Their effect on the atmospheric corrosion of magnesium alloys has drawn more attention due to the higher supply of dissolved CO_2_ in the thin electrolyte film. Magnesium carbonate, formed during atmospheric exposure, is a primary corrosion product and has better protectiveness than the magnesium hydroxide [49]. Similarly, in the physiological environment, due to the presence of a variety of carbonates, their influence on the corrosion rate of Mg alloys has been demonstrated to be profound [50]. The inhibition performance of dissolved bi/carbonate is highly dependent on the electrolyte pH, being more effective at alkaline pH [51], since the concentration of bi/carbonate rapidly decreases in favor of carbonic acid as the pH becomes more acidic. The inhibition effect of individual carbonates seems to be unsatisfactory for severe corrosive conditions, while the synergistic effects with other inhibitors, including Li^+^ ions [51,52] and phosphates (H_2_PO_4_^−^/HPO_4_^2−^/PO_4_^3−^) [50], can boost the overall reduction in corrosion rate. The environmental benignity of the carbonates also renders them suitable and unrestricted components to improve corrosion inhibition.

**Calcium**

**Figure 4:**
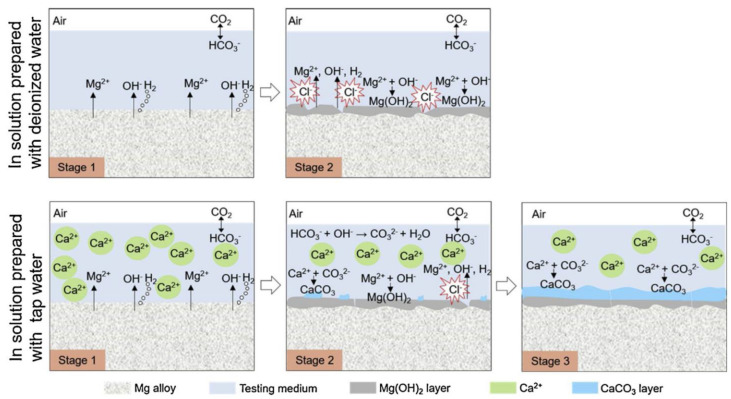
Schematic presentation of the corrosion processes and formation of corrosion products of binary Mg0.5Zn or ternary Mg0.5ZnX (X = 0.5Ca or 0.2Ge) alloys in 0.9 wt.% NaCl solutions prepared with deionized water and tap water (as source of Ca^2+^ and HCO^3−^). Adapted from [55] with permission from Elsevier.

Calcium is a well-known component of phosphate conversion coatings for magnesium alloys (see **PART I** of this review [8,53,54]). Although standalone calcium cation, Ca^2+^, does not look like a typical corrosion inhibitor for magnesium alloys, even a very low concentration of carbonate, characteristic for tap water or carbonate and phosphate found in fresh-water lakes, is sufficient for triggering precipitation of calcium-carbonate (and phosphate) species. Multiple reports refer to a strong corrosion inhibition effect when Ca^2+^ is combined with anions that form sparingly soluble precipitates in an alkaline environment, which is immediately (within the first minute of immersion) generated on Mg interface upon contact with aqueous electrolytes. These ions include phosphates (H_2_PO_4_^−^/HPO_4_^2−^/PO_4_^3−^), carbonates (HCO_3_^−^ and CO_3_^2−^), and fluorides, F^−^ [24,25,50,55,56]. Under the presence of Ca^2+^ cations, the precipitation of several Ca-containing phases such as hydroxyapatite (Ca_5_(PO_4_)_3_OH), CaMg(CO_3_)_2_, and CaCO_3_ occurs on magnesium surface, prompted by the alkalinization front due to cathodic HER and ORR. The formation of these products buffers the interface pH in a range of 7.8–8.5 at room temperature. Figure 4 illustrates the formation of the additional layer of CaCO_3_ on top of Mg(OH)_2_ upon immersing binary Mg0.5Zn or ternary Mg0.5ZnX (0.5Ca or 0.2Ge) alloys in 0.9 wt.% NaCl when tap water was used for solution preparation. In the presence of 59 mg/L calcium at pH 7.48 in tap water, the corrosion resistance of the alloys was improved by almost one order of magnitude, according to the electrochemical test results [55]. Besides, EIS clearly shows the formation of an additional time constant at high frequencies ascribed to an outer layer containing calcium-carbonate (and often phosphate) species [50,55]. Similar to calcium ions, Sr^2+^, Zn^2+^, and Mn^2+^ ions promote precipitation of phosphates on alkaline Mg surface and are used for chemical conversion treatments; see **PART I** of this review [8].

**Vanadates**

Vanadate salts, well known for their corrosion suppressing properties when applied to aluminum [57] and zinc [58] alloys, have also been tested as corrosion inhibitors for magnesium alloys [59]. Feng et al. [59] found that the inhibition mechanism of vanadates applied on AZ31 magnesium alloy differs from that observed for aluminum alloys. Due to the considerable reduction power of magnesium, vanadate oxyanions containing V^5+^ under neutral and alkaline conditions are reduced to V^3+^ and V^2+^ in the short term. Reductive adsorption of vanadium oxoions under acidic conditions results in a film containing V^4+^, also in the short term. However, independent of pH, all the reduced vanadium species are reoxidized back to V^5+^ upon exposure to air. Corrosion inhibition by vanadate is attributed to the formation of a reductively adsorbed layer comprised of vanadium oxides and magnesium hydroxides. Only dense and continuous layers of tetrahedral vanadate speciation, formed under neutral and alkaline conditions, provide reasonable protection. Given the specific chemistry of vanadate oxyanions, they are quite efficient at forming corresponding conversion layers or combined with other protective approaches, such as incorporation in Layer Double Hydroxide (LDH) [8] or Plasma Electrolytic Oxidation (PEO) surface layers [60]; see below in this review.

**Molybdates**

**Figure 5:**
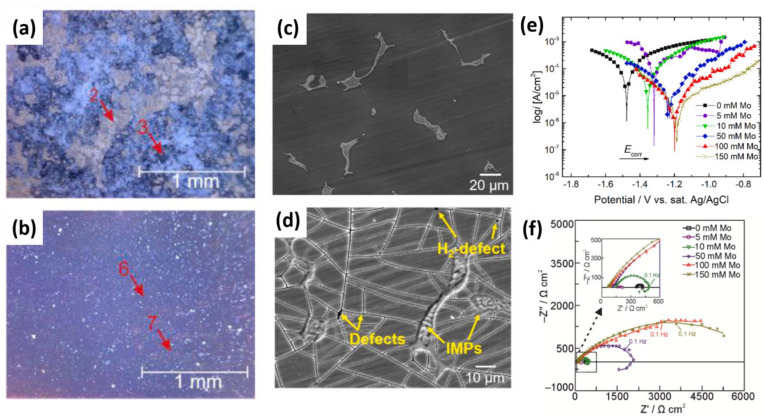
(**a**,**b**) light microscopy images of the WE43 alloy surface after 24 h exposure to 0.05 M NaCl solution without (**a**) and with (**b**) 100 mM of Na_2_MoO_4_ inhibitor. Numbers and arrows indicate the areas of Raman spectroscopy carried out in the corresponding paper [61] (**c**) SEM image of as-polished WE43 surface. (**d**) Surface morphology of the WE43 alloy after 24 h exposure to 0.05 M NaCl solution with 100 mM Na_2_MoO_4_. (**e**) Potentiodynamic polarization curves obtained after 24 h exposure to 0.05 M NaCl without and with molybdate inhibitor. (**f**) Nyquist EIS plots in 0.05 M NaCl solution without and with varying amounts of Na_2_MoO_4_ inhibitor after 24 h of exposure. Adapted from [61] with permission from Elsevier.

Molybdate protective systems have been investigated for Mg alloys, either as conversion coatings (see **PART I** of this review [8]), or as inhibitors incorporated into other coating systems (see Section 5). However, the inhibition effect and mechanism of individual molybdates on bare Mg substrate have not been investigated in depth until recently. Kharitonov et al. [61] investigated the effect of Na_2_MoO_4_ with concentrations of 5–150 mM in 0.05 M NaCl solution on WE43. A significant corrosion inhibition was observed after 24 h for 100 mM concentration of sodium molybdate Figure 5a,b. The formation of a mixed-valence Mo(VI)-Mo(V) polymerized layer was proposed as the inhibition mechanism of molybdate ions [61]. Additionally, the precipitation of brownish hydroxide MoO(OH)_3_ could block the Mg surface. The formation of insoluble magnesium molybdate MgMoO_4_ has also been hypothesized [62].

The Mo-containing precipitates exhibited a high tendency to form on the Mg matrix rather than the second phases Figure 5c,d, which resulted in a significant positive shift of OCP and a reduction in anodic activity of the Mg substrate Figure 5e. Reaching a critical concentration is essential to achieve a high inhibition efficiency, below which the corrosion rate of the Mg substrate is accelerated (Figure 5f). The acceleration occurs due to the partial surface coverage, which in turn may intensify the local corrosion. A very similar high corrosion inhibition performance of Na_2_MoO_4_ was also reported for AZ31 magnesium substrate [63], which may be reflective of its universal effect. Molybdates have low harmfulness to the environment and can be considered a promising substitute for chrome(VI)-based inhibitors.

**Selenite**

Similar to arsenate, selenite (SeO_3_^2−^) can be reduced in the form of crystalline and amorphous Se^0^ on an AZ31 magnesium alloy [64,65]. The reduction reaction to Se^0^ was shown to be not limited only to low pH values, as in the case of arsenate. A strong reduction in cathodic activity was recorded independent of selenite concentration in the range of 1–50 mM. A mixed layer containing MgSeO_3_ hydrate and Mg-Se oxyhydroxide film containing Se^0^ and Se^4+^ was formed on the Mg surface, which led to a significant reduction in the corrosion rate. Similar to arsenate, selenite poses some risks from a human health perspective, and the concentration in use must be kept at low levels for industrial uses [66,67,68].

**Nitrates**

Nitrates are commonly used as corrosion inhibitors for steel. A few reports also showed their significant positive effect on magnesium alloys [10,28,29,30,69]. A universal high inhibition effect of KNO_3_ has been observed by Lamaka et al. [10]. In spite of several auspicious results, the inhibition mechanism is quite untouched. Additionally, as with the RE-based inhibitors, cations in the form of salts of nitrates are frequently studied as corrosion inhibitors, overlooking the effect of nitrate anion itself. It has been suggested that the inhibition mechanism of NO_3_^−^ is not related to the competitive adsorption between NO_3_^−^ and Cl^−^ [29]. Rather, the inhibiting effect of NO_3_^−^ and its reduced form NO_2_^−^ might be related to the interference in Fe^2+^/Fe^3+^/Fe oxidation–reduction–redeposition cycle as one of the main cathodic impurities in most Mg alloys [10]. 

There are still many untouched inorganic systems to be explored as corrosion inhibitors for magnesium and its alloys. Many of them are potentially expected to provide corrosion inhibition effect based on their performance either as corrosion inhibitors for aluminum and its alloys, or as precursors for effective conversion coatings, or as alloying elements in Mg with a low corrosion rate. 

For instance, the corrosion inhibition mechanism of some alloying elements, such as germanium, indium, and arsenic, is partially attributable to the re-deposition of zero-valent forms of the same elements following their dissolution in the corrosive medium during corrosion. The same mechanism has also been proposed for salts of the same elements that act as corrosion inhibitors in solution. However, there are still scanty (if any) reports on the effect of salts of indium and germanium as corrosion inhibitors.

## 3. Organic Inhibitors 

Numerous environmentally benign organic inhibitors have been tested in search of chromate-free inhibitors. Many organic substances with a relatively high ability to reduce the corrosion rate of magnesium alloys have already been reported, while their inhibition efficiency has rarely been compared with that of chromate. The reported effective organic substances range from very simple organic molecules such as salts of formic acid, the simplest carboxylic acid, to very complex molecules such as tannic acid or high-molecular-weight surfactants.

What makes the understanding of the inhibition mechanism of organic substances difficult is that even small variations in the molecular structure and composition of either inhibitor or substrate can significantly change the mechanism of inhibition. The inhibition mechanisms prompted by organic substances are mostly based on forming a complex with either magnesium ions already dissolved into the corrosive solution or atoms located on the magnesium alloy surface. Complex formation occurs by donating one or more electron pairs by organic ligands to a magnesium atom. The atoms in an organic molecule with the ability to donate electron pairs are O, N, S. When the complexation occurs on the surface of magnesium, it results in an adsorptive layer, isolating the magnesium surface from the corrosive medium. On the other hand, when the complexing occurs in the solution with magnesium ions, it must form an insoluble compound that precipitates on the magnesium surface to function as an effective precipitation inhibitor. In the following, the most important and effective groups of organic inhibitors are studied, and current progress in the understanding of their inhibition mechanisms is reviewed.

### 3.1. Adsorption and Precipitation Inhibitors

The inhibition mechanism of several effective corrosion inhibitors for Mg alloys has been ascribed to their ability to be adsorbed on the Mg surface. The adsorption of molecules on the surface can be viewed as forming a barrier layer, preventing corrosive species from reaching the Mg surface. This adsorption mechanism is usually postulated from the inhibitor molecule chemistry, which has electron donor atoms, which can interact with Mg atoms on the surface. However, the direct evidence of such adsorption has been barely reported for Mg alloys. The other effect of such adsorption is changing the morphology of Mg(OH)_2_ as the main corrosion product of Mg corrosion. For instance, the adsorption of organic molecules such as BTA has been claimed to serve as a nucleating agent on the Mg surface, stimulating the precipitation of denser crystalline Mg(OH)_2_ film [70].

**Triazole** and its derivatives, such as 3-amino-, 4-amino-, 3-mercapto-, and 3-hydroxy-, have also been tested as corrosion inhibitors for Mg alloys [10,71,72,73,74,75,76]. Benzotriazole also exists in commercial inhibiting compounds such as Ardrox 3961 [77]. Reported results are controversial, ranging from high corrosion inhibition to dramatically accelerated degradation of Mg alloy. Similarly, it has been shown that the effect of benzotriazole (BTA) and its 5-chloro- derivative differs depending on experimental conditions and tested alloy [10,70,71,75,78,79,80]. Considering the high condition dependency of BTA and its harmfulness to aquatic life, it is not likely to find any industrial application. 

**Schiff base (SB) compounds** are a group of organic substances with at least one double bond between carbon and nitrogen atom -C=N-R (imine; R is either Alkyl or Aryl group). The imine group offers a strong bond with the metallic ions. SBs with polar substituents at suitable molecular positions can form chelates with metal ions, which boosts their bonding strength. The effective inhibitive properties of many Schiff base compounds have been frequently reported for corrosion protection of aluminum [81,82,83], steel [84,85,86], and copper [87,88]. Their inhibition effect is mainly attributed to their adsorption on the metallic surface to form a protective film that isolates the metals from the aggressive environment. The same mechanism of corrosion inhibition is also claimed for Mg and its alloys, although direct evidence for such adsorption has been barely reported. The relatively simple route for synthesizing a countless number of SBs via condensation reactions of aromatic or aliphatic amine and carbonyl compounds [89] renders them a highly potential territory for looking for an effective corrosion inhibitor for Mg alloys. SBs are widely used in bio-applications such as antimicrobial, anti-inflammatory, antiviral, and anticancer agents, which implies their low environmental toxicity [89]. Nevertheless, there are only a few reports of SBs for magnesium; many of them have been tested in acidic conditions or in ethylene glycol aqueous solutions, which are not common conditions for the industrial application of magnesium [90,91,92].

**Figure 6:**
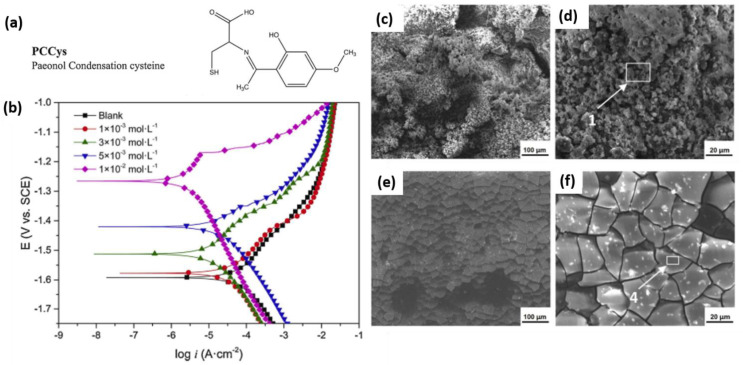
(**a**) Chemical structure of PCCys Schiff-based molecule. (**b**) Potentiodynamic polarization curves for Mg-Zn-Y-Nd alloy in 0.9 wt.% NaCl solution with and without different concentration of PCCys. (**c**–**f**) Corrosion morphologies of Mg-Zn-Y-Nd alloy after 7 days immersion in 0.9 wt.% NaCl solution: (**c**,**d**) without PCCys, (**e**,**f**) with 10^−2^ M PCCys. Numbers and arrows in (**d**,**f**) indicate the areas of EDS analysis carried out in the corresponding paper Adapted from [93] with permission from Elsevier.

Recently, Ma et al. [93] synthesized three different SBs molecules via a condensation reaction of paeonol with amino acids tyrosine (PCTyr), phenylalanine (PCPhe), and cysteine (PCCys). All the SBs in the concentration range of 10^−3^–10^−2^ M could inhibit the corrosion of a Mg-Zn-Y-Nd alloy in 0.9 wt.% NaCl solution, among which PCCys (see the molecular structure in Figure 6a) at a concentration of 10^−2^ yield the highest inhibition efficiency of 92%. A strong reduction in the anodic activity of the Mg alloys was observed for all the SBs (Figure 6b), reflecting their anodic-type inhibition. The morphology of the corrosion products has been changed in the presence of the SBs in the electrolyte, Figure 6c–f, which was postulated as the precipitation of insoluble complexes between Mg^2+^ and the SBs. The possibility of a precipitation mechanism has also been postulated in other works; however, direct evidence of this was not provided. 

In addition to the adsorption of inhibitors, they can form insoluble complexes with magnesium ions that precipitate and block the magnesium surface. Of course, their tendency to form a bond with magnesium atoms can still occur on the surface of magnesium; however, experimental results show that a precipitation mechanism is more likely to happen than direct adsorption of this group of organic substances on the magnesium substrate.

**Figure 7:**
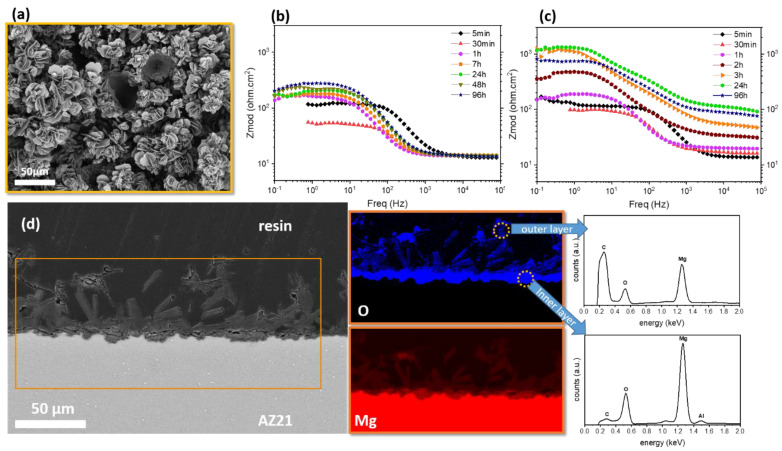
(**a**) Surface morphology of bare AZ21 exposed to 3.5 wt.% NaCl electrolyte containing 8HQ. (**b**,**c**) Bode plots of the EIS spectra obtained for the AZ21 Mg during 4 days of immersion in NaCl 3.5 wt.% solution with and without 8HQ, respectively. (**d**) Cross section view of (**a**) along with the elemental mappings of the marked area. Adapted from [102] with permission from Elsevier.

**8-Hydroxyquinoline** (8HQ) is a strong complexing agent that has the ability to chelate with Mg^2+^ and form an insoluble complex, which is highly stable in the pH range of 9.4 to 12.7 [94]. It is actually used in classical analytical analysis as a low-selectivity group reagent due to its ability to form insoluble chelates with a number of cations. At pH > 8, apart from Mg^2+^, the following cations precipitate with 8HQ: Ca^2+^, Al^3+^, Fe^3+^, Cr^3+^, Co^2+^, Ni^2+^, Zn^2+^, Cd^2+^, Cu^2+^, and Ag^+^. Several reports of strong corrosion inhibition by 8HQ on different magnesium alloys confirm that 8HQ is a plausible candidate for commercial use on Mg parts [25,95,96,97,98]. On the other hand, some reports [10,80,99,100] suggest that 8HQ and its derivatives have a minimal or negligible inhibition effect on magnesium alloys, notwithstanding critical factors related to testing conditions, such as the substrate elemental composition and microstructure, the composition and concentration of testing electrolyte, concentration of inhibitors, initial solution pH, the ratio between electrolyte volume and exposed magnesium surface area, and eventually the testing time. Generally, the corrosion inhibition effect of 8HQ is attributed to the formation of an insoluble complex with Mg^2+^ ions (8HQ(Mg)), which precipitates and blocks the surface of magnesium substrate [25,95]. Although the flower-like morphology of 8HQ(Mg) complex (Figure 7a) may provide some degree of hydrophobicity [101], a continuous blocking layer seems to be achieved with delay. This is evident in the slow increase in Mg AZ21 impedance exposed to a 8HQ-containing corrosive NaCl electrolyte (compare Figure 7b,c) as well as rather dispersed 8HQ(Mg) flakes present on the already formed corrosion product layer after 1 day of immersion (Figure 7d).

**Figure 8:**
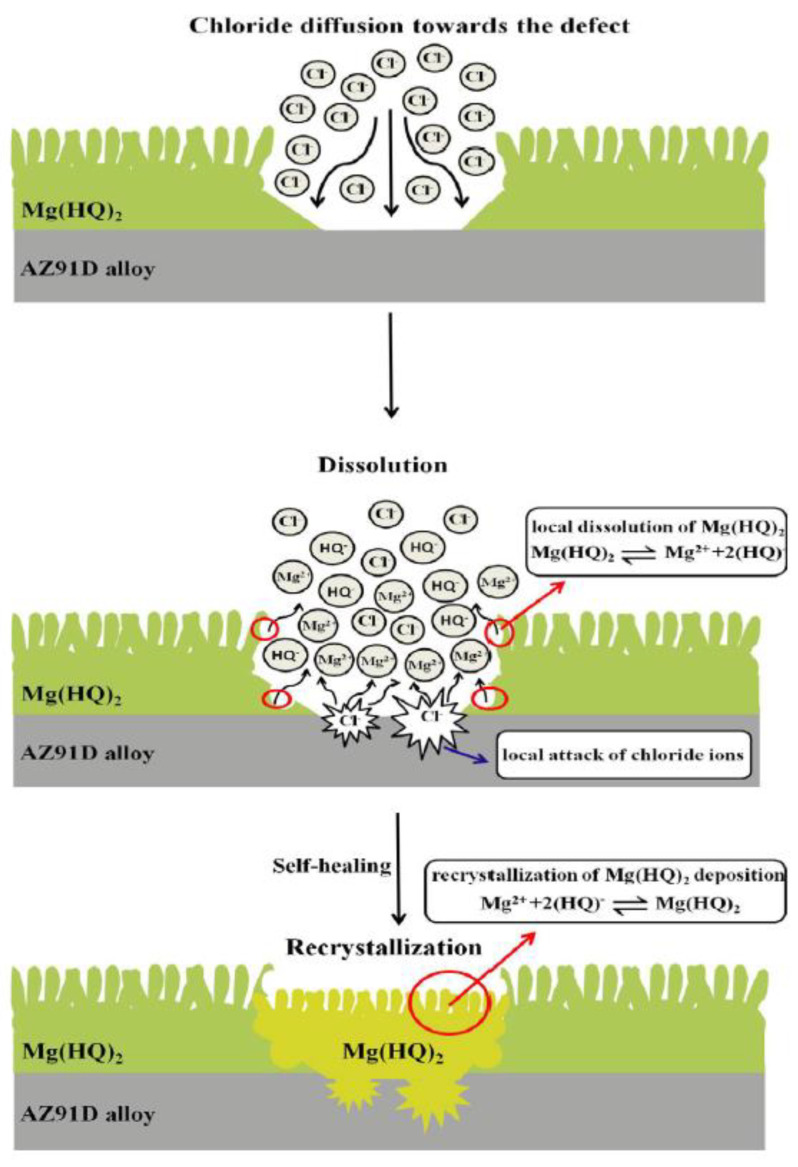
Schematic illustration of inhibition mechanism of 8HQ(Mg) layer on a Mg substrate. Note: Mg(HQ)_2_ in the figure and 8HQ(Mg) in the text refer to the same complex. Adapted from [96] with permission from Elsevier.

A layer of 8HQ(Mg) precipitating on a Mg substrate is believed to provide self-healing properties [96]. The corrosion of a Mg substrate at a defect leads to a locally high concentration of Mg^2+^ and OH^−^. The latter causes the partial dissolution of 8HQ(Mg) flakes, which re-precipitates in the local enriched Mg^2+^ region on the coating defect (see Figure 8 for the schematic mechanism of self-healing by 8HQ(Mg)).

Although the effectiveness of 8HQ as a corrosion inhibitor for Mg has resulted in numerous previous and recent related research activities [103,104,105], the risk of mutagenicity and chemical instability in light hinders the wide use of 8HQ in commercial applications.

### 3.2. Surfactants 

Surfactant (the short form of surface active agent) is a general term used for a group of organic molecules that are conventionally described as a single hydrocarbon tail connected to an ionic or polar head group [106]. Many surfactants are already used in various industries to control the corrosion rate of steel and Al engineering parts [2]. Similar to other organic inhibitors, surfactants interact with magnesium atoms, mostly by forming bonds through their electron donor atoms, N, O, and S. For surfactants, electron donation occurs mostly on the metal surface, resulting in their predominant adsorptive characteristic. Therefore, surfactants are able to cover the metal surface and act as a barrier to corrosive media. The barrier effect is intensified when the complex of surfactants with Mg cation is highly insoluble and precipitates along with surfactant adsorption. Moreover, a surfactant molecule consists of one (or more) long organic chain (also called a “tail”) that brings two main enhancements to the inhibitive characteristics of this group of chemicals:

1. Due to the hydrophobic properties of the tail of surfactants in aqueous environments, they can effectively keep corrosive media from coming into contact with the magnesium surface.
2. Due to the bulky tail of surfactants, they are highly effective at covering surfaces, even in small concentrations.

It has been shown that as the length of the surfactant chain increases (when other factors are equal), higher inhibition efficiency is achieved [107,108], mainly due to the fact that the abovementioned inhibitive characteristics become more effective [109]. However, the inhibition efficiency reduces when the chain length exceeds a certain value [110,111]. Steric hindrance prevents the bulky molecules from staying close to each other and covering the entire surface. Moreover, the chain length affects the electronegativity of the head group in the surfactant, which influences the adsorption ability of the surfactant molecule on the surface. Furthermore, the long surfactant tail limits its solubility in aqueous media.

**Figure 9:**
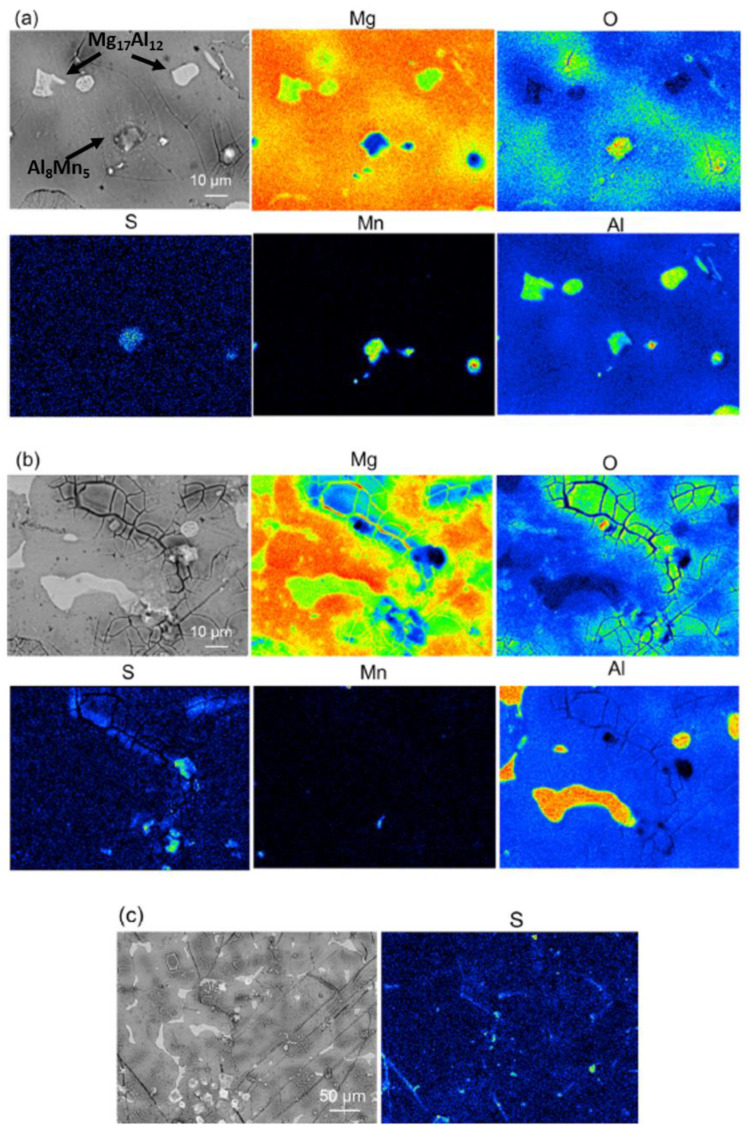
Main elemental distribution (Mg, O, S, Mn, and Al) on Mg surface after immersion for 6 h (**a**) and 48 h (**b**,**c**) in 3.5 wt.% NaCl solution with addition of 0.05M SDS. Adapted from [116] with permission from Elsevier.

Changes in the head of surfactants, which function as the adsorptive part of the surfactant, can significantly change the inhibition efficiency. Various surfactants with the molecular structure of carboxylic head linked to alkyl chains of different lengths have been studied as corrosion inhibitors for Mg alloys [10,75,80,109,112,113,114,115]. They are environment-friendly organics that are mostly derived from natural sources. For instance, lauric acid (C12) and myristic acid (C14) are mainly found in human breast milk and coconut milk. Stearic acid (C18) and palmitic acid (C16) are widely used in the production of soaps and detergents. Most of the alkyl carboxylates were reported as mixed-type inhibitors with a small shift in the corrosion potential to noble values. However, chemisorption of the surfactant’s head on regions that are characterized by cathodic behavior can effectively improve the protective performance of inhibitors on magnesium alloys. Adsorption of surfactants on phases with cathodic characteristics is particularly effective when low d-orbital energy elements such as Zr and RE are present in the chemical composition of the magnesium alloy [80,112]. Analogously, the preferential adsorption of sodium dodecylsulfate (SDS) on the Mn-containing second phase Al_8_Mn_5_ rather than on Mg_17_Al_12_ in AZ91 Mg alloy has been detected by [116], as shown in Figure 9. However, possible preferential adsorption of SDS on Mg(OH)_2_, which forms more on the Mn-containing secondary phase with a higher galvanic potential difference to the Mg matrix [117,118], should also be taken into consideration.

**Figure 10:**
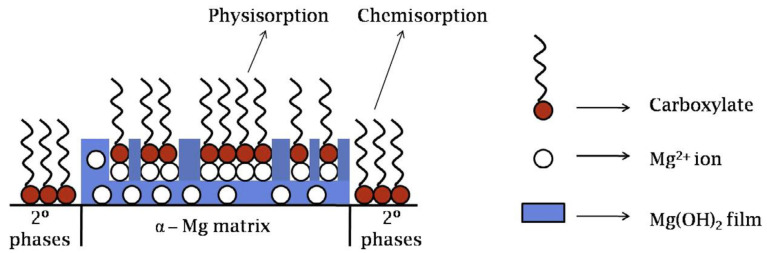
The schematic representation for the surface adsorption of alkyl carboxylates over different phases of ZE41. Adapted from [112] with permission from Elsevier.

Figure 10 is a schematic representation of the surface film formation on ZE41 alloy immersed in an aqueous solution containing alkyl carboxylates. The formed surface film consists of a complex of alkyl carboxylate with magnesium ion, Mg(OH)_2_, and chemisorbed surfactant, dominantly on the second phases with higher cathodic characteristics with respect to α-Mg phase [112]. It is believed that anodic inhibition of magnesium alloys is achieved mainly through densification of the porous surface Mg(OH)_2_ film by physical adsorption of Mg carboxylates [115] rather than the direct adsorption of alkyl carboxylate anions on magnesium surface. 

**Figure 11:**
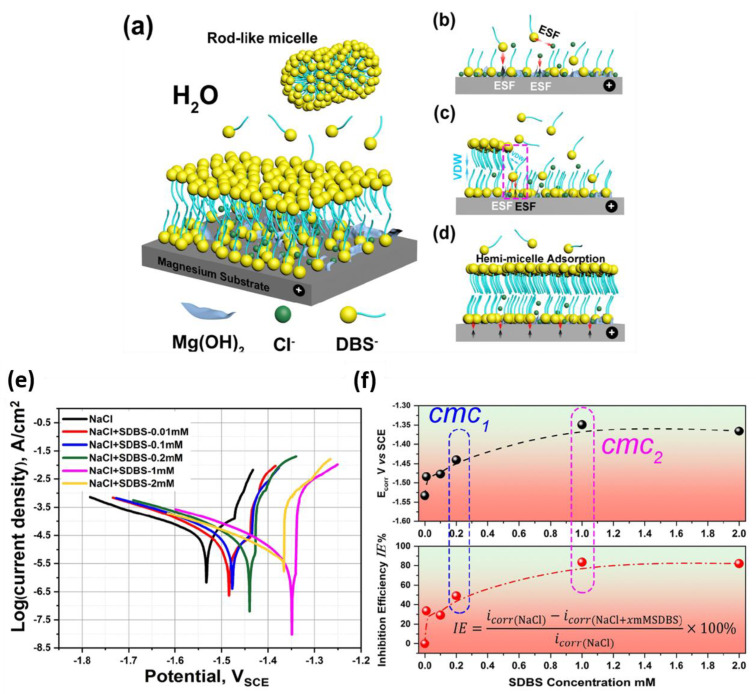
(**a**–**d**) Adsorption model of Mg surface in NaCl+SDBS solution with high SDBS concentration. (**a**) Bilayer structure formed by DBS^−^ surfactant. (**b**) Negatively charged DBS^−^ and Cl^−^ adsorb on positively charged Mg substrate via electrostatic force (the black and white marked “ESF”, respectively, represent electrostatic repulsion and attraction). (**c**) Competitive adsorption between DBS^−^ and Cl^−^ (VDW illustrates “van der Waals” force) (**d**) Formation of hemi-micelle adsorption film. (**e**,**f**) Tafel potentiodynamic polarization measurements. (**e**) Tafel curves recorded in different solutions. (**f**) Corrosion potential and inhibition efficiency. The labeled cmc_1_ and cmc_2_ represent the concentrations of SDBS reaching spherical and rod-like micelles, respectively. Adapted from [128] with permission from Elsevier.

Sulfates and sulfonates are other functional groups as the head-group of surfactants. Sodium lauryl sulfate [119], N-lauroyl-N-methyltaurine [119], sodium N-lauroylsarcosine [10,119], sodium dodecylsulfate (SDS) [116,120,121,122,123], and sodium dodecylbenzenesulfonate (SDBS) [10,25,80,95,119,124,125,126,127,128] have been studied as inhibitors for magnesium alloys, among which SDS and SDBS have drawn more attention. In addition to oxygen and sulfur atoms with lone-pair electrons, the benzene ring is also able to share its lone-pair electrons with magnesium atoms on the surface. Due to its chemical composition and molecular structure, SDBS is considered a good adsorptive organic chemical. Adsorption of SDBS molecules on the surface is combined and reinforced by precipitation of SDBS-Mg complex that further blocks corrosion active sites [119,128,129]. Chen et al. [128] proposed an inhibition mechanism model based on the competition between DBS^−^ and the aggressive Cl^−^ ions to be adsorbed on the Mg surface. They suggested that the inhibition efficiency of the SDBS increases with the concentration until it exceeds the concentration required to form a bi-layer (hemi-micelle adsorption) of DBS^−^ molecules covering the Mg(OH)_2_ nano-sheets on the Mg surface (Figure 11a–d). This trend was also confirmed by the inhibition efficiency extracted from the dynamic polarization test (Figure 11e,f). 

Recently, Li et al. [121,122] have claimed that the presence of SDS in a NaCl electrolyte exposed to AZ91 and AM50 Mg alloy can facilitate the oxidation reaction of the Mg matrix with the dissolved O_2_ in the electrolyte. As a result, a relatively thick passivating MgO layer can form on the Mg matrix, significantly reducing the anodic activity of the substrate. This claim was confirmed by the nine-fold increase in the corrosion rate in the same electrolyte after deaeration by purging with N_2_ gas. However, the chemical mechanism of such enhancement in the MgO formation has remained unanswered.

In general, the high corrosion inhibition ability of surfactants at reasonable cost effectiveness can be reflected in their common use in commercial inhibition products, which are also effective for Mg alloys [77,130].

### 3.3. Ionic Liquids

**Ionic liquid (IL)** is a type of salt with a low melting point below 100 °C [131], which is mostly composed of organic cation–anion pairs [132,133]. High conductivity (0.1–14 mS/cm), broad electrochemical stability windows, high thermal stability, and low vapor pressure [134] are some of the unique physicochemical properties of ILs. In addition, the organic nature of the cation–anion pair allows innumerable formulations of the ILs, which can also be “designed” according to their desired properties. Several reports have investigated the use of ILs to form a conversion coating on Mg alloys (**PART I** of the review). However, only recent studies (mainly published after 2020) by a few scientific groups have investigated the application of ILs as corrosion inhibitors for Mg alloys [135,136,137,138,139,140,141,142]. 

So far, only bis(trifluoromethanesulfonyl)imide (NTf_2_, also abbreviated as TFSA in the literature) has been the most used anion for ILs as Mg corrosion inhibitors; while the effect of different cations, including pyrazolium [135,136,138], imidazolium [137], and phosphonium [139,140,141,142] have been investigated. The role of NTf_2_ anion in IL inhibitors plays an important role in forming a protective layer on the Mg substrate. The decomposition of NTf_2_ into smaller fragments, including F^−^ ion, leads to the formation of a thin protective MgF_2_ film. The presence of MgS and MgSO_4_, as the result of the reaction of Mg ions with fragmented NTf_2_, has also been concluded from XPS analysis [137,138]. Although the decisive role of cations in ILs can be clearly reflected in their different corrosion protection performances, the mechanistic view of their inhibition roles is not clear and is usually explained by the different degrees of adsorption of their fragments on the Mg surface. 

The concentration of investigated ILs is usually below 1 mM, with an increasing inhibition efficiency with their concentration. A relatively high (~87%) inhibition efficiency (calculated based on a potentiodynamic test) has been reported for 1 mM 1-decyl-3-methylimidazolium bis(trifluoromethylsulfonyl)amide ([DMIm][NTf_2_]) in rigorous 3.5 wt.% NaCl solution on an AZ31 alloy [137].

In a recent work by Kurchavov et al. [143], the surface activity of a CP-Mg and AZ61 alloy was investigated in two IL-water mixture solutions, in which the ILs were composed of acetate (OAc^−^) anion and two different 1-methyl-pyrrolidiunium-based cations, denoted as BMPyrr^+^ (1-bytyl-) and mPEG_n_MPyrr^+^ (1-methoxy-polyethylenglycol-). Their difference is that the former contains an aliphatic chain, while the latter contains an etheric chain of polyethylene glycol. Interestingly, although Mg alloys dissolved in these electrolytes at relatively high anodic potential and current, the HER rate on both magnesium substrates was significantly reduced at anodic polarization (up to +1 V vs. E_oc_) only when mPEGnMPyrr^+^OAc-H_2_O (rather than BMPyrr^+^OAc-H_2_O) was employed. This was explained by formation of low adherent acetate-containing passivating film. 

The limited recent reports of the remarkable effects of ILs as “green” corrosion inhibitors for Mg alloys can be taken as a promising approach to finding an effective substitute for chromate-based inhibitors. Nevertheless, it is at its nascent stage and deserves further investigation.

### 3.4. Iron Binding Inhibitors 

Most of the organic inhibitors described above target the anodic dissolution of magnesium. Inhibition of cathodic activity is another possibility for suppressing corrosion. The detrimental effect of iron impurities on the acceleration of magnesium corrosion via cathodic activation has been known for decades [144] and keeps attracting the attention of many scientific groups [145,146,147]. In a recent explanation of this phenomenon proposed by Höche et al. [147], the cathodic activity of the corroding front can be increased over time (which means a higher galvanic corrosion rate) by re-deposition of iron ions that originated from already detached iron-rich impurity particles from the surface and dissolved into the electrolyte. The negative difference effect (NDE), which is the acceleration of cathodic hydrogen evolution reaction at anodic polarization, has also been explained by such re-deposition of iron. Michailidou et al. [148] and Mercier et al. [149] supported this mechanism by providing new experimental evidence. Based on this explanation, Lamaka et al. [150] showed that the prevention of iron re-deposition by strong iron complexing agents can effectively reduce the corrosion rate of commercially pure magnesium. Moreover, Vaghefinazari et al. [151] have clearly shown that the NDE can be suppressed by the presence of the strong complexing agent EDTA on a CP-Mg. Following this concept, numerous organic inhibitors have been found with inhibition efficiencies comparable to or exceeding that of chromium (VI) [10].

In particular, derivatives of salicylic and pyridinedicarboxylic acids were found to have the highest inhibiting efficiency for a number of commercially important alloys (AZ31, AZ91, AM50, WE43, ZE41, and Elektron 21) and three grades of pure magnesium. 

Corrosion of most of the tested magnesium materials were strongly inhibited by amino-, methyl-, thio-, nitro-, and dinitro-derivatives of salicyliate. The suppression of iron re-deposition was accounted for the observed high inhibition effects. In contrast with the aforementioned derivatives of salicylate, sodium salts of salicylic itself and sulfosalicylic had weak or even adverse inhibition effects for on the tested alloys, which is due to the formation of stable and soluble complexes with Mg^2+^ ($logK_{Mg^{2+}}^{st}$ = 5.1 for sulfosalicylic acid) [152]. In this instance, the positive effect of iron complexation is counteracted by the accelerated dissolution of magnesium. Amino-, methyl-, nitro-, and dinitro-derivatives of salicylic acid also form stable complexes with Fe^3+^ ($logK_{Fe^{3+}}^{st}$ = 18.13 for 3-methyl-, $logK_{Fe^{3+}}^{st}$ = 14.57 for 5-methyl-, $logK_{Fe^{3+}}^{st}$ = 14.19 for 3-nitrosalicylic acid [152,153,154]). The highest inhibiting effect of salicylic acid derivatives was found for highly corroding pure Mg with active iron impurity particles and aluminum-containing alloys AZ31, AZ91, and AM50. The formation of stable and soluble complexes between Mg^2+^ and two derivatives of benzoic acids, namely 4-hydroxybenzate and 3,4-dihydroxibenzate ($logK_{Mg^{2+}}^{st}$ = 9.84 for 3,4-dihydroxybenzoic acid), also resulted in a high dissolution rate of tested Mg materials, in spite of their chelating effect with Fe^3+^ [153]. Salicylaldehyde demonstrated high inhibiting efficiency for RE-containing alloys but should not be considered for industrial use due to its toxicity to aquatic life [10,155,156]. 

Sodium salts of several derivatives of pyridinedicarboxylic acids, (2,6-, 2,5-, 2,3- and 3,4-PDCA) showed high inhibition efficiency (more than 70% on average) for all tested magnesium materials. PDCAs are also able to form strong chelates with Fe^2+/3+^ (e.g., for 2,6-PDCA $logK_{Fe^{3+}}^{st}$= 17.13 [157]); thus, their observed high corrosion inhibition effect was partially attributed to the suppression of iron re-deposition. Sodium salts of derivatives of pyridine with only one carboxylic acid group, namely picolinic and nicotinic acids, could also weakly inhibit most of the test Mg materials. 

**Figure 12:**
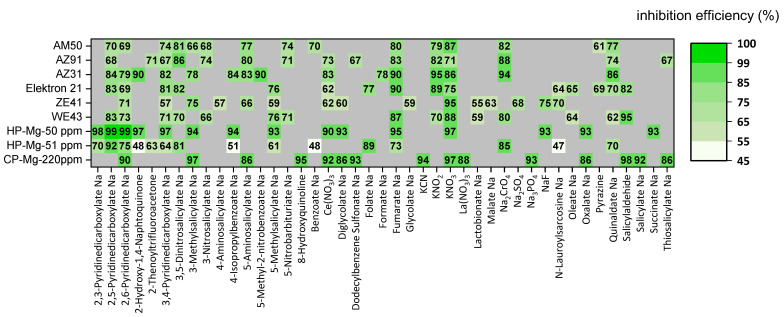
Inhibiting efficiency of top 15 corrosion inhibitors for pure Mg, RE, and Al containing Mg alloys. Based on the data presented in Table 5 of reference [10].

Figure 12 provides an overview of the most efficient corrosion inhibitors as derived from hydrogen evolution measurements in pH neutral 0.5 wt.% NaCl electrolyte with or without corrosion inhibitor. A corrosion inhibitor that is found to be effective for RE-containing Mg alloys is not always effective for Al-containing Mg alloys, though it is more likely to be effective if it is applied within the same group of Mg alloys.

**Figure 13:**
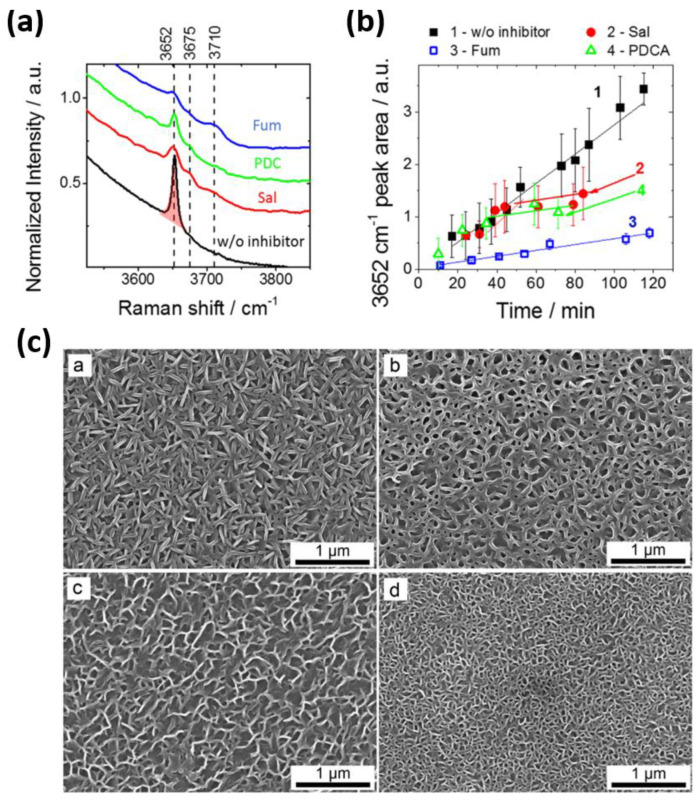
(**a**) Fragments of in situ Raman spectra recorded on CP-Mg342 in 0.1 M NaCl in absence and in presence of corrosion inhibitors; (**b**) growth kinetics of Mg(OH)_2_ recorded on CP-Mg342 in 0.1 M NaCl with or without the corrosion inhibitors; (**c**) SEM micrographs of CP-Mg342 surface after 30 min contact with flowing 0.1 M NaCl solution with and without 0.05 M inhibitors (**a**: without inhibitors; **b**: sodium salicylate (Sal); **c**: 2,5-pyridin-dicarboxylate (PDCA); **d**: fumarate (Fum)). Adapted from [158] with permission from Elsevier.

One of the valuable outcomes of the comprehensive screening of corrosion inhibitors for different magnesium alloys by Lamaka et al. [10] was the discovery of chemical substances that are able to effectively inhibit six tested alloys and three grades of pure magnesium, which can be considered “universal” inhibitors. Among them, PDCs, fumarate, and derivatives of salicylates were further investigated in several reports at different experimental conditions and using different characterization methods to gain more insights into their inhibition mechanisms [158,159,160,161,162]. Apart from their role in suppressing the iron re-deposition of these inhibitors, it was found that the complex formation between 2,5PDC and Mg^2+^ precipitates as a coordination polymer, further protecting the Mg substrate. The corrosion inhibition of 2,5PDC requires an initial dissolution of Mg substrate, which was evident from the induction period in rate of Mg(OH)_2_ growth measured by in situ Raman characterization of the Mg surface (Figure 13a,b). Salicylate is also able to stabilize the Mg(OH)_2_ nano-crystals via a chemisorption mechanism. Similar to 2,5PDC, salicylate requires an initial dissolution of Mg in order for its inhibition to become activated. Adsorption of fumarate on the metallic Mg or MgO leads to an immediate inhibition effect.

Moreover, all of the abovementioned universal inhibitors affect the morphology of Mg(OH)_2_ as the main corrosion product, resulting in a more compact and less porous microstructure (Figure 13c), which in turn hinders the penetration of corrosive species. A recent work by Cui et al. also emphasizes the effect of organic molecules on the nucleation and growth of Mg(OH)_2_, which can determine their corrosion inhibition efficiency [163]. 

### 3.5. In Silico Screening of Corrosion Inhibitors

Another valuable outcome of the comprehensive screening of 150 organic corrosion inhibitors for different magnesium alloys by Lamaka et al. [10] is that it created a relatively large database of inhibiting efficiency values, acquired under the same conditions. These coherent data allowed extension of the data-driven computational methods, such as machine learning [164,165,166,167], to the field of magnesium corrosion inhibition [168,169,170,171]. The successful predictions of several new corrosion inhibitors for Mg alloys via in silico methods have already been reported in several recent papers [168,170]. There are technically a limitless number of organic compounds potentially effective at inhibiting the corrosion of metals. Experimental approaches are capable of evaluating only an insignificant percentage of such infinite chemistry space. Thus, *in silico* methods have recently come into play to expand the explored chemical space and predict the effective corrosion inhibitors for Mg alloys [168,169,170,171,172,173]. 

Although time consuming (months scale) at the stage of methodological development of robust and reliable models, data-driven computational methods can explore large chemical spaces much more quickly, thus saving resources and labor while not generating any waste. In a recent paper by Würger et al. [168], the accuracy of the developed model was tested by screening a commercial database containing 7094 organic compounds (provided by Thermo Fisher Scientific). Experimental testing of such scale is unrealistic, as it would require years of continuous experimenting, even employing high-throughput methods and associated costs of millions of euros. This *in silico* screening resulted in the prediction of a number of molecular structures, which were proven to be rather strong corrosion inhibitors. 1,2,4-benzenetricarboxylate and two nitro-phthalates were shown to possess inhibiting efficiency above 70% through experimental testing. The similarity-based discovery workflow is available online at www.exchem.de; accessed on 2 December 2020. Other examples of predicted (and experimentally validated) inhibitors for a commercial purity magnesium are sodium salts of 0.05 M of trimesic acid and n-Octyl-gallate with inhibition efficiencies of more than 80% in 0.5 wt.% NaCl solution [170].

With the fast development of computational methods, software, and computational power, computational algorithms are able to rapidly process the experimental databases to train machine learning models. The lack of coherent and reliable experimental databases, covering large chemical spaces, for data-hungry artificial intelligence approaches remains a problem. 

The most valuable datasets are those acquired under the same experimental conditions so that the data in the set are directly comparable. This, however, limits the further extension of such models to other magnesium alloys, inhibitor concentrations, etc. Additionally, the quickly growing amount of corrosion inhibition efficiency values, acquired by a large variety of methods, for different substrates (Al-, Zn-, Cu-alloys and steels along with Mg), with different experimental conditions and testing times, calls for efficient ways to organize them and possibly find the most efficient “universal” corrosion inhibitors with a broad window of conditions where such inhibitors remain highly efficient. Galvão et al. [174] have established an online database of corrosion inhibitors, which are freely available for contributors datacor.shinyapps.io/cordata/; accessed on 8 May 2021. It enables a better overview, centralized access, and simultaneous yet independent methods of data processing, possibly yielding valuable general trends.

**Figure 14:**
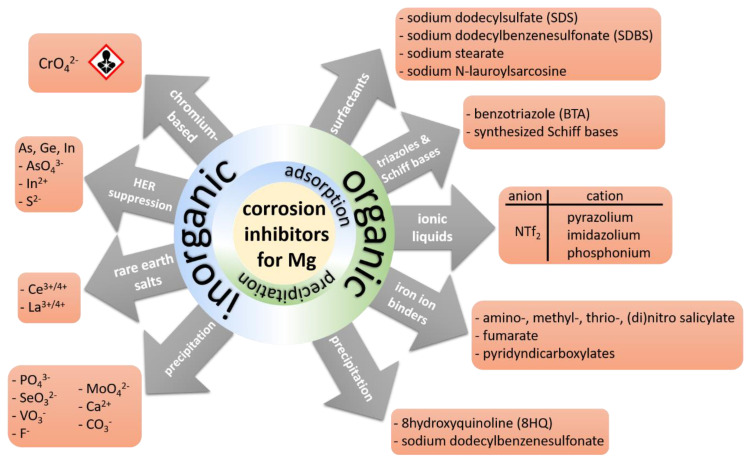
General overview of different categories of corrosion inhibitors for Mg and its alloys with their most effective examples.

An overview of different organic and inorganic categories of corrosion inhibitors for Mg and its alloys is presented in Figure 14. The following sections focus on the strategies to boost the inhibition effect via mixing individual inhibitors from different categories and incorporating individual and mixtures of inhibitors into various coating systems. 

## 4. Inhibitor Mixtures: Synergistic Inhibiting Effect

The combination of individual inhibitors in order to boost the overall inhibition performance by a synergistic effect is a simple and promising approach. Synergy occurs when the inhibitive property of the combination exceeds the sum of the individual components’ performance. The synergistic combinations of inhibitors cannot be predicted a priori because the behavior of synergies is usually non-linear. In spite of requiring extensive testing and combinatorial optimization [175], inhibitor mixtures are a promising research direction. 

It should be noted that many already developed inhibitors are actually a combination of two components, yielding a synergistic inhibition effect, however, the impact of one component is usually overlooked. For instance, when nitrates of organic and inorganic cations are studied, the effect of nitrate on the corrosion of Mg is usually neglected, and the observed inhibition is fully attributed to the cation. 

Synergic effect of two well-known inhibitors, SDBS and 8HQ, on AZ91D was investigated by Gao et al. [95]. Although the stand-alone 8HQ could achieve a 95% inhibition efficiency (calculated by data extracted from EIS) after 72 h of immersion in ASTM D1384-87 corrosive solution, with the addition of SDBS, the highest inhibition efficiency of 98% could be achieved after a shorter time of 48 h. Note that the presence of sodium bicarbonate in the ASTM D1384-87 has been neglected in this work, in spite of its reported inhibition impact (see Section 2.5).

**Figure 15:**
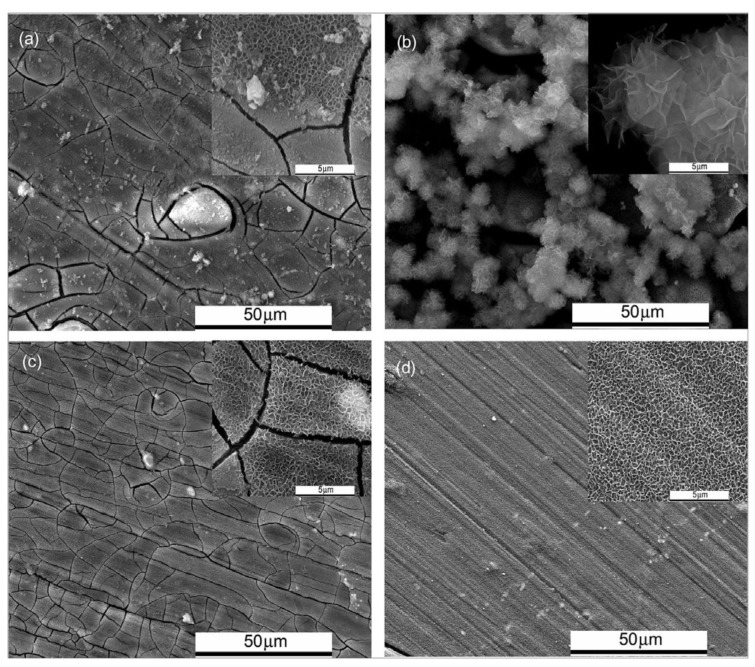
SEM micrograph of the surface of GW103 alloy after 24 h of immersion in (**a**) blank solution; (**b**) blank solution + 0.1 mM zinc nitrate; (**c**) blank solution + 0.5 mM APTS–Na and (**d**) blank solution + 0.5 mM APTS–Na + 0.1 mM zinc nitrate [176]. “Blank solution” is a corrosion testing solution from ASTM D1384, which consists of corrosion test solution: 184 mg/L Na_2_SO_4_ + 138 mg/L NaHCO_3_ + 165 mg/L NaCl. Adapted from [176] with permission from Elsevier.

Hu et al. [176] investigated the inhibition effect of organo-silicate aminopropyltriethoxysilicate (ATPS-Na) accompanied by inorganic zinc nitrate on Mg-10Gd-3Y (GW103). Although each of these inhibitors shows a weak inhibition effect, their combination boosts the corrosion protection. The improvement is attributed to the remarkable reduction in the size of the cracks in the formed film on the substrate. This is the result of the co-deposition of zinc and magnesium organo-silicate. Figure 15 presents SEM micrographs of the surface of GW103 alloy after immersion in a solution containing both inhibitors individually and in combination. Nevertheless, the effect of NO_3_^−^ in zinc nitrate and bicarbonate in the blank corrosive solution has been overlooked again.

Alginic acid and its derivatives have been widely used as environmentally friendly corrosion inhibitors for some engineering metals such as steel [177,178,179] and aluminum [180,181,182]. Few works also have reported the limited effectiveness of sodium alginate (SA) additives in corrosive inhibition of magnesium alloys [183]. The main issue of using SA as corrosion inhibitor is its inherent massive molecule, which is likely to face steric hindrance when it is adsorbed on the surface, preventing the formation of a compact film on magnesium alloy [184]. Li et al. [185] added sodium alginate to a 3.5 wt.% NaCl at concentrations between 0 and 0.05 M, while two other inhibitive substances, sodium tungstate, and sodium silicate, were already present at fixed concentrations. The EIS results revealed that the presence of SA could significantly improve the inhibition efficiency up to 94% for 0.03 M SA. At the same time, excessive SA addition can almost completely negate this positive effect. They suggested that at SA concentrations lower than 0.03 M, SA is adsorbed on the defects of the already formed passivation composite film of MgWO_4_ and MgSiO_3_. However, at higher concentrations, the preferential adsorption of SA on the surface hinders the deposition of silicate and tungstate, causing deterioration of their corrosion inhibition characteristics. These results emphasize the fact that achieving the highest synergistic inhibition effect from individual inhibitors may depend on finding a sweet spot of mixture concentration; any deviation from this composition may have an adverse effect. 

In another recent work, Hou et al. [184] investigated the combined inhibitive effect of SA at a fixed concentration of 0.05 wt.% and sodium phosphate at a concentration range of 0.05–0.2 wt.% on AZ31. Thermodynamic studies on the formation of insoluble Mg_3_(PO_4_)_2_, and SA-Mg complexes showed that SA-Mg complex is the dominant component of the film covering the AZ31 surface under the initial stage of immersion. Then, deposition of insoluble Mg_3_(PO_4_)_2_ modifies this film as the immersion time is extended, and corrosion of Mg substrate results in the formation of a more protective complex film consisting of Mg(OH)_2_, SA-Mg complex and Mg_3_(PO_4_)_2_. Sodium phosphate has also been coupled with other organic inhibitors, including sodium dodecylsulfate (SDS) [186], sodium dodecylbenzene sulfonate (SDBS) [80], and 2-mercaptobenzothiazole (MBT) [187], with the aim of achieving a synergistic inhibition effect. The synergistic effect of these combinations has been attributed to the formation of an adsorption layer on the Mg_3_(PO_4_)_2_/Mg(OH)_2_ film and Mg substrate. The highest synergistic effect strongly depends on the sweet spot in the ratio of two components to assure the sufficient precipitation of Mg_3_(PO_4_)_2_ and adsorption of the organic component.

**Figure 16:**
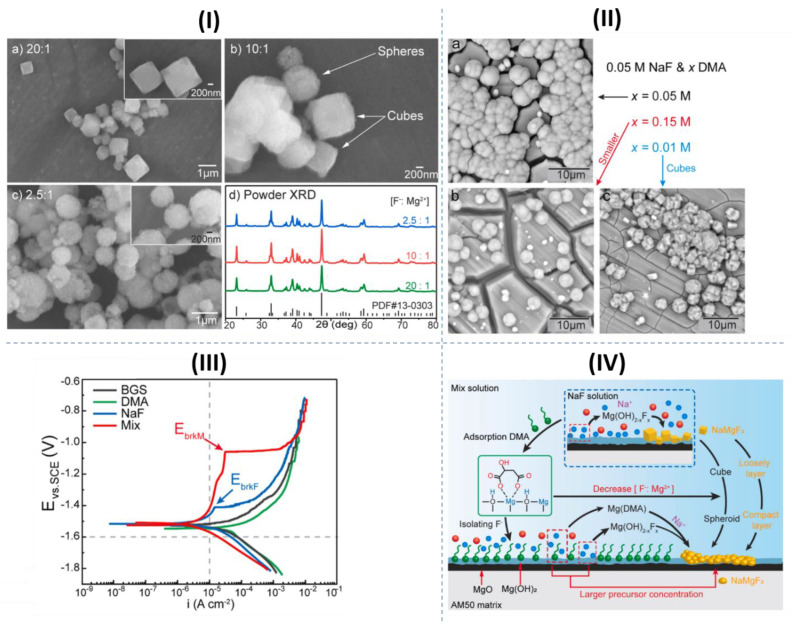
(**I**) The morphology of NaMgF_3_ particles synthesized at varied reactants (NaF: MgCl_2_) molar ratios of (**a**) 20:1, (**b**) 10:1, (**c**) 2.5:1, and (**d**) the XRD patterns of the synthesized particles. (**II**) Morphology of NaMgF_3_ particles obtained from Mix solutions containing 0.05 M NaF and different molar concentrations of DMA (**a**) 0.05 M, (**b**) 0.15 M, and (**c**) 0.01 M. (**III**) Potentiodynamic polarization curves of the AM50 specimen immersed in the NaCl background solution (BGS) with and without 0.05 M DMA, 0.05 M NaF, and Mix after 24 h of stabilization. (**IV**) Schematic illustration of the synergistic corrosion inhibition mechanism of NaF and DMA hybrid inhibitor. Adapted from [45] with permission from Elsevier.

Recently, Qui et al. [45] investigated the synergistic corrosion inhibition of NaF and DL-malate (DMA) on a Mg-Al-Mn magnesium alloy. The DL-malate molecules chemisorb on Mg(OH)_2_/MgO film through binding of the carboxylate groups with Mg sites, which itself, to some degree, hinders the penetration of corrosive Cl^−^ ions towards the substrate. On the other hand, precipitation of MgF_2_ and NaMgF_3_ as the result of the interaction of Mg^2+^ ions with F^−^ ions is a well-known inhibition mechanism of fluoride-based inhibitors. Indeed, the concurrency of these two inhibition mechanisms may boost Mg corrosion mitigation. However, the synergistic corrosion inhibition was rationalized based on an additional factor, which is the modification of the morphology of NaMgF_3_ precipitates with DMA molecules. Figure 16I shows the morphology of NaMgF_3_ particles synthesized with different ratios of NaF:MgCl_2_. Decreasing the F^−^:Mg^2+^ ratio leads to the modification of NaMgF_3_ particles from cubic to spherical form. The same variation in the morphology of the precipitated particles on the Mg alloy was observed when the concentration of DMA in the corrosion electrolyte increased (Figure 16II). Thus, it was postulated that DMA could regulate the F^−^:Mg^2+^ ratio by forming a complex with Mg^2+^, hindering them from interacting with F^−^ ions. As a result of the precipitation of spherical NaMgF_3_ particles with higher efficacy to cover the surface, a significantly enhanced passivation of the Mg surface could be achieved (Figure 16III). The schematic illustration of the synergistic corrosion inhibition mechanism of NaF and DMA can be seen in Figure 16IV.

## 5. Combining Inhibitors with Other Corrosion Protection Strategies

The application of corrosion inhibitors onto metallic surfaces can be accomplished in a number of ways. If the metallic part is used in continuous contact with an aqueous electrolyte, the simplest way to deliver the inhibiting substances to the corroding region is to load the corrosive medium with the inhibitor. For example, in an engine cooling system, the coolant contains inhibitors to protect the part from premature failure due to corrosion. However, in most applications, the metallic parts are not fully submerged in a liquid medium and experience only periodic contact with a corrosive electrolyte or thin condensation electrolyte films. In such a case, the corrosion inhibitors can be incorporated into the coatings applied onto the metallic substrate. 

The addition of corrosion inhibitors to the coating system can impart active inhibition properties and improve overall protective performance, provided that the coating’s barrier properties are not adversely affected by the inhibitors. Furthermore, the interaction between the inhibitors and the coating’s components may lead to a (partial) loss of the active capacity of the inhibitor. One of the promising ways to incorporate inhibitors into coatings is to encapsulate the corrosion inhibitor in a container that is ideally fully compatible with the inhibitor, coating matrix, and substrate. Several types of repository systems for inhibitor substances have been described in the literature over the last decade [188]. Repository systems can be classified by their composition (e.g., nano-containers with polyelectrolyte shells, polymer shells, layered double hydroxides, mesoporous inorganic materials, etc., reviewed in [189]) or by the release mechanism (e.g., mechanical rupture, desorption, ion exchange, pH, or ΔE -controlled release, etc., reviewed in [190]). The following section reviews several technologies relevant to magnesium alloys, enabling loading protective coatings with corrosion inhibitors.

**Sol–gel** is an effective and economical coating technique that is environmentally friendly, can be applied onto complex shapes, and has a low process temperature [191]. Additionally, sol–gel-based systems provide good adhesion to metallic surfaces and top coats, which are subsequently applied to the cured sol–gel [192]. Loading sol–gel matrix with corrosion inhibitors has been reported as a highly effective strategy to enhance the corrosion protection properties of the coated magnesium alloys. 

Galio et al. [193] added 8HQ inhibitor into a sol–gel coating formulation deposited on an AZ31 magnesium alloy at different stages of sol–gel synthesis: after and before hydrolysis of the sol–gel precursors. Higher corrosion protection was observed when 8HQ was incorporated after the hydrolysis. They postulated that the addition of the inhibitor in the sol–gel formulation before the hydrolysis step can partly deactivate the 8HQ, while the already formed silane-based network after hydrolysis is more inert to 8HQ molecules. The improvement in the corrosion protection properties of the sol–gel coating was attributed to the formation of a complex between the 8HQ and the Mg^2+^ during the corrosion of the Mg substrate. The insoluble 8HQ(Mg) blocks the micropores and cracks of the sol–gel coating, which results in enhanced barrier properties of the coating. 

Toorani et al. [194] incorporated 5 ppm of four different organic inhibitors, namely 8HQ, indole-3-carbaldehyde (I3C), 2-mercaptobenzoxazole (MBO), and sodium diethyldithiocarbamate (DDTC), into a sol–gel with the mixture of γ-amino propyltriethoxysilane (APS) and γ-glycidoxypropyltrimethoxysilane (γ-GPS). The modified sol–gel mixture was applied on a PEO-coated AZ31 substrate. All the inhibitors showed adverse or negligible effects on the barrier properties of the sol–gel coating except for 8HQ. The initial corrosion resistance of the system modified by 8HQ (evaluated by low-frequency impedance values) was comparable to the unmodified system. However, a sharp increase in the charge transfer resistance value after 6 h of immersion indicated an active inhibition characteristic. As a result, the incorporation of 8HQ into the sol–gel coating significantly improved the overall corrosion resistance of the system.

The modification of silane-based sol–gel coatings with cerium ions has been extensively studied with the aim of improving the corrosion protection of different metallic substrates. The barrier properties of a silane coating are improved by the addition of cerium in the sol formulation owing to several proposed mechanisms. The incorporation of Ce^4+^ ions into the silane network as a substitution for Si atoms leads to a more packed and interconnected silane network [195]. Moreover, the higher rate of sol bath acidification due to the presence of Ce accelerates the hydrolysis process [196,197]. Furthermore, cerium ion remnants inside the sol–gel micropores can form insoluble hydroxides and block the pathways for the penetration of the corrosive medium towards the substrate. This improvement in the barrier properties of the sol–gel coating may further intensify during the corrosion of Mg due to the generation of more OH^−^ as a result of cathodic reaction on the Mg substrate [195,197]. In one of the early studies on the incorporation of Ce in sol–gel coatings, Montemor and Ferreira [195] observed considerably weaker anodic and cathodic current densities using SVET over an artificial defect made on a silane coating doped with cerium ions. The active corrosion inhibition was claimed to be caused by the release of cerium ions and their precipitations in the form of hydroxides on the bare Mg. It should be noted that the incorporation of Ce^3+^ ions into the sol–gel coating is often conducted using the nitrate salt of cerium. However, as previously mentioned, the inhibition effect of nitrate ion itself on the corrosion of the Mg substrate [10] is often ignored. 

**Figure 17:**
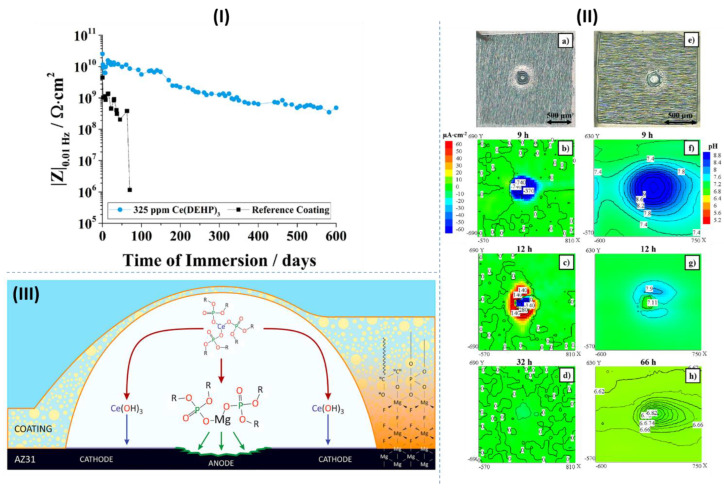
(**I**) Evolution of low-frequency impedance modulus (0.01 Hz, obtained through EIS) for reference and Ce(DEHP)_3_-modified coatings during immersion in 0.05M NaCl. (**II**) Optical micrographs, and SVET (**b**–**d**) and SIET (**f**–**h**) maps obtained for different immersion times of the Ce(DEHP)_3_-modified coating in 0.05M NaCl. Optical micrographs correspond to the beginning of immersion for SVET (**a**) and SIET (**e**) measurements. (**III**) Schematic mechanism of action of Ce(DEHP)_3_ corrosion inhibitor added to the hybrid epoxy-silane coating for protection of magnesium alloy substrate. R represents the hydrocarbon chain. Adapted from [200] with permission from Elsevier.

One drawback of cerium-based corrosion inhibitors is their high solubility in neutral aqueous environments, which may result in their high release rate and early leaching from a coating [198]. The application of organic anions, such as phosphates, to bind to cerium cations can control their solubility in the aqueous solution [199]. Recently, inspired by the same approach, Calado et al. [200,201] synthesized a cerium Tri(bis(2-ethylhexyl)phosphate) (Ce(DEHP)_3_) inhibitor. Then, an epoxy-silane coating modified by 325 ppm Ce(DEHP)_3_ was applied on AZ31 and WE43 magnesium alloys. The barrier properties of the coating on AZ31 alloy were significantly improved, resulting in three orders of magnitude higher impedance value (modulus of impedance at 0.01 Hz) after 600 days of immersion in 0.05 M NaCl compared to the non-modified coating after just 70 days of immersion, Figure 17I. The local pH increase due to the cathodic reactions on the magnesium substrate leads to the hydrolysis of Ce(DEHP)_3_, resulting in the release of Ce^3+^ cations and organophosphate anions. The Ce^3+^ precipitates on the catholically active area as cerium hydroxides. On the other hand, the organophosphates can form a hydrophobic layer at the anodic area or make stable complexes with Mg^2+^. The combined effect of released Ce^3+^ and organophosphate ions leads to “self-healing” properties, as observed on an artificially defected area on the coated Mg substrate via SVET and SIET measurements (Figure 17II). The schematic mechanism of corrosion inhibition and self-healing is illustrated in Figure 17III.

Incorporated cerium does not always show good compatibility with the sol–gel matrix [197,202,203]. For instance, the incorporation of cerium nitrate into a sol–gel network at a concentration higher than 0.6 wt.% resulted in the formation of network defects in the sol–gel matrix. The formed micro-holes retained water, facilitating the penetration of corrosive substances through the sol–gel coating [204]. The formation of structural defects in the sol–gel matrix by increasing the concentration of doped Ce^3+^ has also been observed elsewhere [205]. 

The inefficacy of the barrier properties of sol–gel coatings due to the incompatibility of the sol–gel matrix with the incorporated inhibitors can be overcome by loading nano/micro containers with good compatibility with the sol–gel coating, and then incorporating them into the coating [206,207,208,209,210,211,212]. Thus, the detrimental interaction of the inhibitors with the sol–gel is minimized. For instance, in a recent work, Adsul et al. [206] loaded the Ce^3+^/Zr^4+^ ions into halloysite nanotubes, which were capped with polymeric microcapsules. Then, the loaded nanotubes were dispersed into a silica sol–gel and deposited on AZ91 magnesium alloy. A significant increase in the sol–gel coating resistance over 24 h of immersion in 3.5 wt.% NaCl was reported. 

It is of essential importance that the terms “active protection” and “self-healing” be properly used when the effect of an inhibitor incorporated into a coating system is under investigation. Initial stronger barrier properties of a coating with incorporated inhibitors do not necessarily mean that it is able to be actively involved in the suppression of the corrosion process on the substrate. Attempts to incorporate several amino acid inhibitors into different sol–gel coatings have resulted in significant improvement in the barrier properties of the coating on Mg alloys [202,213,214,215], yet the active inhibition effect of the incorporated inhibitors is not evidenced. Note that the “intrinsic self-healing” ability of sol–gel coatings, which does not require the addition of corrosion inhibiting agents, is another well-known approach to achieving “self-healing” properties in sol–gel coatings [216]. In this case, the “healing” properties involve reversible chemical reactions in the sol–gel network that lead to the closure or sealing of damage in the coating.

**LDH**

The application of Layered Double Hydroxides (LDH) in corrosion protection systems has been one of the most active topics under scrutiny due to their unique anion-exchange properties (see **PART I** of the review [8]). LDHs are generally composed of stacking of positively charged mixed metals M^I^/M^II^-M^III^ hydroxide layers, balanced by the presence of anions [217]. Organic and inorganic inhibitors in their anion form can be intercalated within the LDH inter-galleries and can be released into the corrosive medium in exchange for the corrosive Cl^−^ ions. Therefore, there is a higher control on the inhibitor release rate, which results in long-term active corrosion protection rather than fast leaching of the inhibitor. Furthermore, the trapping of the corrosive Cl^−^ ions mitigates the severity of corrosion at local conditions. It is of importance to identify whether the inhibitors are successfully intercalated into the LDH inter-galleries to benefit from the controlled release functionality or physically trapped within its porous morphology. This can be checked by observing a shift in the diffraction angle of characteristic peaks (003 and 006) [218], as a result of the change in the LDH layer’s distance, accommodating anions with different ionic sizes.

LDHs in a powder/slurry form can be incorporated into a coating system to act as nano-containers for desired inhibitors; or they can be grown directly on the metal surface as a conversion coating (see **PART I** of this review [8]). Inhibitors can be intercalated into LDH inter-galleries during the formation process or post-treatment via an anion-exchange process. 

Different inorganic inhibitors such as molybdate [62,219], vanadate [219,220,221,222], phosphate [219], and nitrate [220] have been successfully intercalated into LDH conversion coatings on Mg alloys. The corrosion inhibition of CO_3_^2−^ on Mg alloys has been reviewed in Section 2.5, can also be intercalated into LDH structure. However, the higher stability of intercalated CO_3_^2−^ compared to Cl^−^ ions [223,224] leads to an inefficient release of CO_3_^2−^ during the immersion in Cl^−^-containing electrolyte. Ce^3+^ ion can be inserted into the LDH structure as a trivalent cation, rather than being intercalated. In such a case, self-healing properties have been postulated when the LDH coating is degraded, and Ce ions precipitate as cerium hydroxide [221,225].

Phytic acid, aspartic acid (ASP) [226,227], lauric acid (LA) [226], 8HQ [228,229], sodium dodecyl benzene sulfonate (SDBS) [230], sodium alginate [230], and 2-mercaptobenzothiazole (MBT) [230] are examples of organic inhibitors that are successfully intercalated in LDH conversion coating on Mg alloys. Deprotonation of mentioned organic molecules, which is usually carried out with NaOH solution, is required to obtain their anion form, so that they can be intercalated within the LDH layers [217]. The increased d_(003)_-spacing between the LDH layers by hosting large anions of organic inhibitors facilitates the anion exchange process with aggressive Cl^−^ [226].

**Figure 18:**
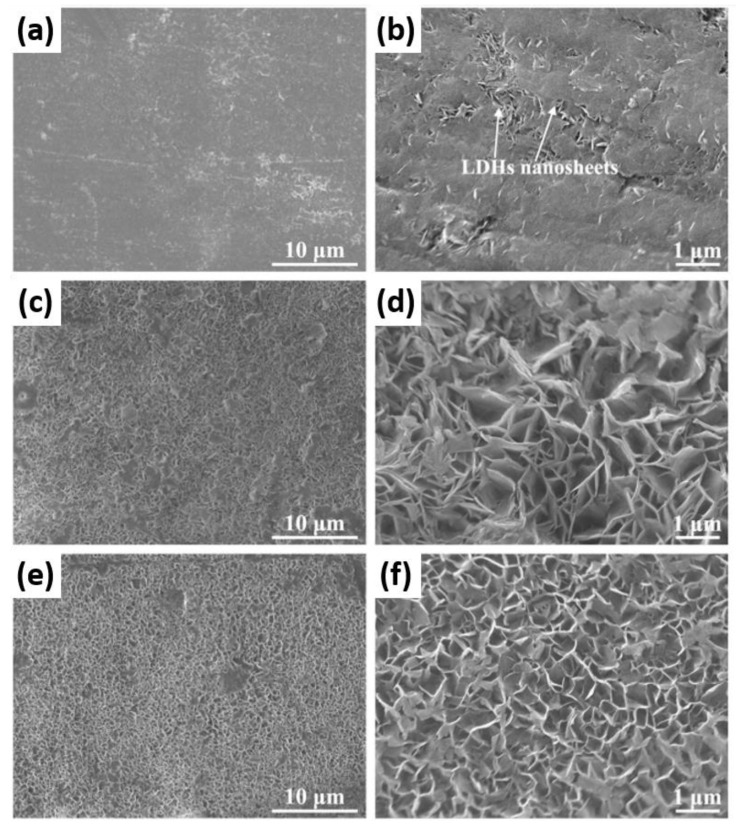
SEM surface micrographs of (**a**,**b**) MgAl-NO_3_^−^-LDHs, (**c**,**d**) MgAl-MoO_4_^2−^-LDHs, and (**e**,**f**) MgAl-SDS-LDHs. Adapted from [231] with permission from Elsevier.

Numerous remarkable enhancements in the anti-corrosion performance of LDH coatings on Mg alloys by hosting inhibitors have been reported during the recent decades. The enhancement of the corrosion resistance is frequently attributed to the sole active inhibition by the inhibitors. However, it should be taken into account that the intercalation of inhibitors into the LDH structure, either via a post anion exchange method or directly during the LDH formation, can affect the LDH morphology, thickness, and porosity, which in turn influence the corrosion protection properties of the LDH layer. For instance, Figure 18 illustrates how the intercalation of sodium dodecyl sulfate (SDS), NO_3_^−^, and MoO_4_^2−^ yield different morphologies of MgAl-LDH flakes on Mg alloy. 

**Figure 19:**
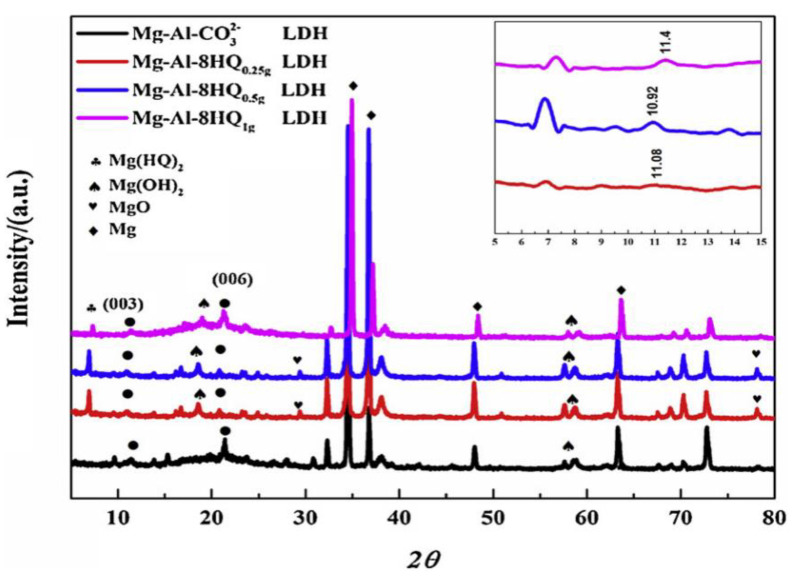
XRD patterns of AZ31 with Mg-Al-CO_3_^2−^ and Mg-Al-8HQ_xg_ (x = 0.25, 0.5 and 1) LDH coatings. Adapted from [228] with permission from Elsevier.

Moreover, the adsorption of surfactants on LDH flakes during the intercalation step endows the specimen surface with a high hydrophobicity, which in turn, enhances the overall corrosion protection performance [232,233] and does not necessarily yield active corrosion properties. Furthermore, the hosted inhibitors may react or form a complex with the LDH precursors during the intercalation process. For example, Anjum et al. [228] intercalated 8HQ inhibitor molecules into a Mg-Al-LDH during the hydrothermal growth of LDH on a AZ31 alloy. The shift of the diffraction peak of (003) plane at the lower 2θ angles indicates the intercalation of anions of 8HQ molecules into the LDH structure, Figure 19. However, the relatively strong peak at 2θ of 6.8° reveals a considerable amount of 8HQ(Mg) complex precipitation [102], which can already fill the pores of LDH and improves its corrosion resistance.

Although the application of LDH in the corrosion field is labeled with “active” and “self-healing”, such functionalities have rarely been evaluated, and presuming an effective inhibition of the intercalated inhibitors is commonly postulated as a necessary “active” corrosion inhibition. Nevertheless, the anion-exchange rate between the intercalated inhibitor and Cl^−^ ion determines the local concentration of anions, and thus the inhibition efficiency of the inhibitor.

**PEO**

Plasma electrolytic oxidation (PEO) coatings can be used as containers for inhibitors owing to their porous structure that provides a potentially high-capacity reservoir for corrosion inhibitors. Incorporation of inhibitors into the PEO pores is usually carried out after the coating process. This is due to the fact that process parameters such as high local heat generation and high-energy arc discharge formation are likely to alter the molecular structure or oxidize the inhibitor, especially in the case of organic inhibitors.

**Figure 20:**
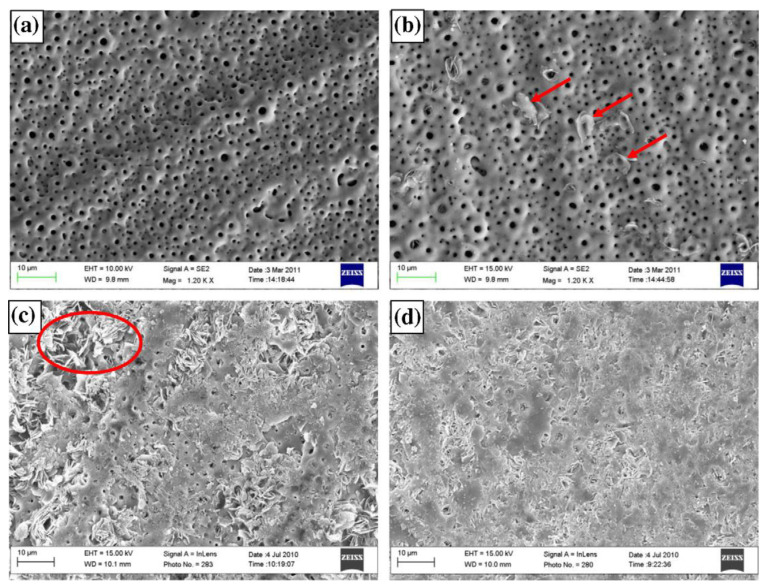
Surface morphologies of anodic films formed in the solutions of 10 g/L NaOH and 18 g/L Na_2_SiO_3_ (**a**) without 8-HQ, with addition of (**b**) 2 g/L 8-HQ, (**c**) 5 g/L 8-HQ and (**d**) 8 g/L 8-HQ under current density 40 mA/cm^2^, frequency 2000 Hz, duty cycle 20% and anodizing time 3 min. Adapted from [234] with permission from Elsevier.

In situ addition of organic/inorganic substances into the PEO bath has been carried out in many scientific works, but their impact on corrosion behavior can be primarily due to their influence on PEO coatings morphology rather than their inhibitive characteristics themselves [234,235,236,237,238]. For instance, the addition of 2 g/L of 8HQ in a PEO bath, containing NaOH and Na_2_SO_4_, results in better uniformity of pores, with a lower number of pores per area in the PEO coating on AZ91 (Figure 20a,b). Some leaf-like particles also appears (indicated with arrows in Figure 20b) with the addition of 8HQ. However, a further increase in 8HQ concentration results in more heterogeneous morphology, with some regions porous and others compact (Figure 20c,d) [234]. A comprehensive review of the effects of organic/inorganic additives to the PEO electrolytes for Mg alloys has been provided in **PART II** of this review [9]. One of the very first attempts to incorporate inhibitors into a PEO layer during the coating formation on a magnesium alloy in a PEO bath was carried out by Blawert et al. [239]. They reported that the addition of chromate to the PEO bath, apart from its undesirable environmental issues, does not exhibit any improvement in the corrosion resistance of the substrate.

**Figure 21:**
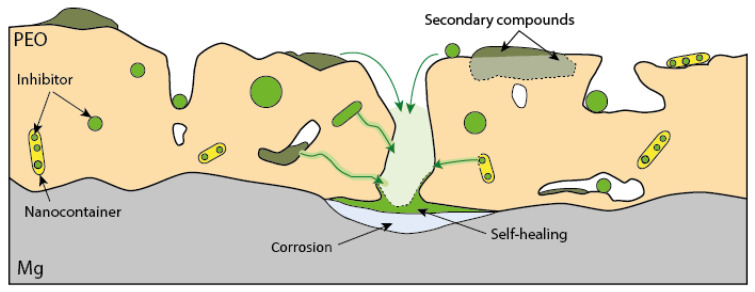
Schematic illustration of the corrosion protection mechanisms provided by PEO coating with inhibitors.

Incorporation of the inhibitors into the formed PEO layer has two main positive contributions to the corrosion protection properties of the coated substrate: 1—corrosion inhibition functionality on the Mg substrate when the PEO coating is damaged or corroded. 2—Blocking the pores of the PEO coating triggered by the substrate corrosion to provide a more compact barrier between Mg substrate and corrosive medium via either adsorption of the organic molecules themselves or via the formation of secondary compounds such as insoluble hydroxides or metal complexes. A schematic illustration of the corrosion protection mechanisms provided by PEO coating with incorporated inhibitors is shown in Figure 21. 

Sealing of the PEO pores after the inhibitor loading is essential to prevent the wasteful release of the inhibitors into the corrosive medium. Sealing of the pores can be achieved via different surface post-treatments that are described in detail in **PART II** of this review [9]. Some chemicals known for their inhibition effect on Mg alloys, such as rare earth element salts [240,241,242,243,244,245,246,247,248,249], phosphates [242,250], stannate [240], and surfactants [240], can be used primarily for the sealing post-treatment. In such a case, their inhibition effect on the Mg substrate also contributes to a reduction in the corrosion rate and provides active inhibition. 

**Figure 22:**
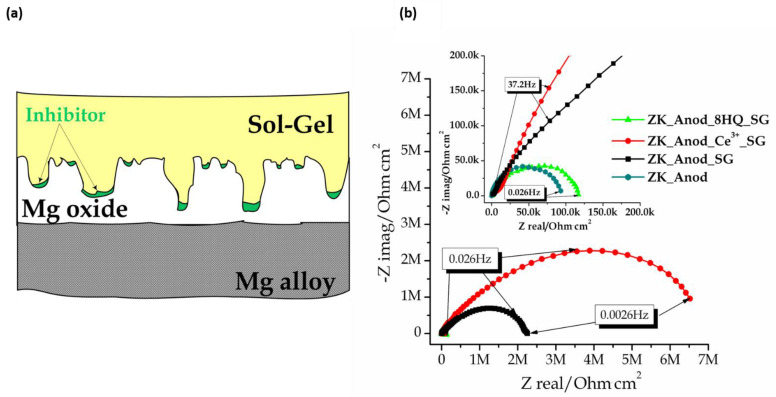
(**a**) Schematic representation of the composite protective coating comprising the highly porous anodized or PEO layer enriched with corrosion inhibitors and sealed with the organic coating; (**b**) Nyquist plots of corresponding ZK30 Mg alloy samples enriched with Ce^3+^ or 8HQ after 2 weeks in 0.05 M NaCl solution. Adapted from [100] with permission from Elsevier.

One of the very first attempts to incorporate inhibitors in PEO coating on Mg alloy, followed by sealing of the PEO pores with an organic top layer, was made by Lamaka et al. in 2009 [100]. Cerium nitrate and 8HQ were incorporated in the porous PEO layer by simple post-immersion in inhibitor-containing solutions, then dried and sealed with a thin sol–gel coating (3–4 µm) synthesized from (3glycidoxypropyl)-trimethoxysilane and titanium(IV) iso-propoxide. Figure 22 illustrates the composite corrosion protection coating based on a PEO coating, loaded with corrosion inhibitors, and sealed with a thin sol–gel layer. Results showed that the incorporated cerium nitrate has good compatibility with the silane-based sol–gel coating that improves its barrier effect, which was reflected in the resistance of the sol–gel layer measured via EIS. Stable and insoluble cerium hydroxides are formed due to the consumption of OH^−^ produced by the cathodic reactions on magnesium. These cerium hydroxides can further improve the barrier properties of the coating complex by sealing the pores of the PEO layer. Moreover, an active corrosion inhibition was observed via SVET on an artificial defect on the coating loaded with cerium. On the other hand, incompatibility of 8HQ with a silane-based sol–gel as the sealing topcoat caused its impairment. Since this pioneering study, the incorporation of 8HQ and Ce-based chemicals into the PEO coating on magnesium alloys with different approaches has been frequently investigated [251,252,253,254]. 

For instance, Gnedenkov et al. [252] loaded 8HQ into a PEO coating on MA8 alloy via simple immersion in a saturated 8HQ bath. Local current density was measured via SVET over an artificial defect in the PEO coating immersed in 0.05 M NaCl. Loaded 8HQ can be activated by pH increase on cathodic sites due to its higher solubility at elevated pH, and then precipitated again in the form of 8HQ(Mg) complex on anodic sites. As a result, the measured anodic local current density on the defected zone was considerably reduced. The increase in the concentration of the 8HQ solution and the duration of the incorporation period enhances the overall corrosion resistance of the system [255]. However, 8HQ is known to form insoluble complexes with Mg^2+^, which can be supplied not only from the Mg substrate, but also from the dissolution of the Mg-containing phases in a PEO coating [102]. As a result, the incorporation step can lead to the sealing of the PEO pores, which improves the barrier properties of the PEO even before 8HQ comes to play as an inhibitor. 

**Figure 23:**
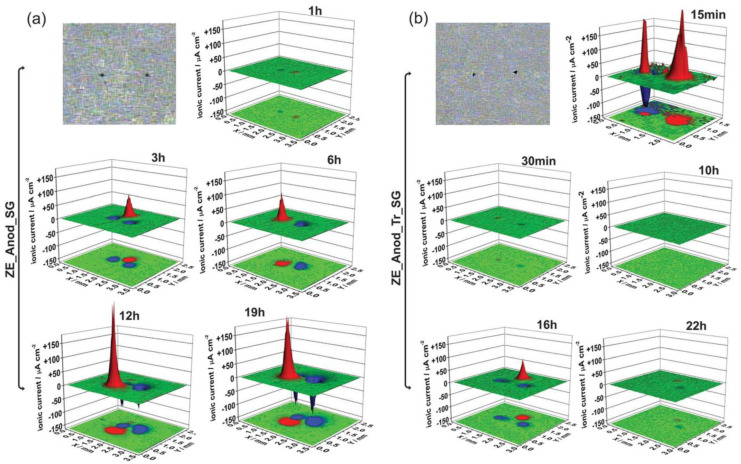
Microphotograph of scanned area and distribution of ionic currents (measured by SVET) for the ZE41 magnesium alloy coated with composite film after different immersion time in 0.05 M NaCl solution (**a**) without loaded 1,2,4 triazole and (**b**) with loaded 1,2,4 triazole. Adapted and reprinted from [76].

A similar concept of multilayer coating was applied on ZE41 magnesium alloy, loaded with 1,2,4-triazole inhibitor and covered by a sol–gel layer [76]. The self-healing properties of this multilayer coating were evaluated using SVET, measuring the local current density over two artificial defects on the coating (with diameter of 200 µm each), Figure 23. An intensive corrosion attack was observed at the beginning of immersion in 0.05 M NaCl solution for the sample loaded with the inhibitor. However, after 30 min, the measured current density reduced to a negligible value, showing active corrosion protection of the coating. No corrosion activity was observed for the next 10 h of immersion. However, after 16 h of immersion, filiform corrosion was observed next to the artificial defects, which was gradually suppressed within the next 6 h [76], indicating active corrosion inhibition. On the other hand, when the coating was not loaded by the inhibitor, considerable ionic currents were firstly detected after 3 h of immersion in the NaCl solution. Then, the detected currents were gradually intensified during the immersion measured.

**Figure 24:**
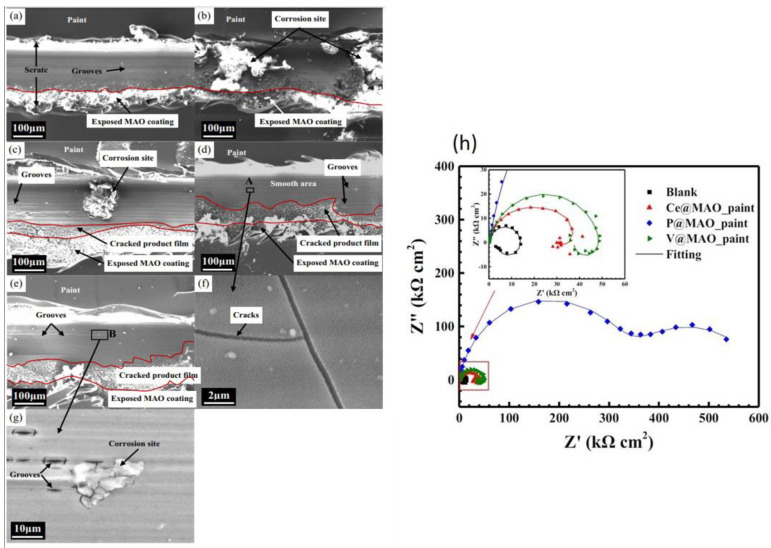
(**a**) SEM micrographs of the scratched areas of a PEO coating on AM60 alloy sealed with a water-based paint; (**b**) after immersion in 3.5 wt.% NaCl for 24 h (**c**) with Ce(NO_3_)_3_, (**d**,**f**) with Na_3_PO_4_, (**e**,**g**) with NaVO_3_. (**h**) EIS spectra of the scratched composite coatings after immersion in 3.5 wt.% NaCl for 24 h. Adapted from [256] with permission from ECS.

Liu et al. [256] compared the self-healing properties of a PEO coating on AM60 alloy loaded with Ce(NO_3_)_3_, Na_3_PO_4_, and NaVO_3_ by observing the surface of an artificial scratch made on the coating during the immersion in 3.5 wt.% NaCl solution, Figure 24. The scratched area of the specimen without any loaded inhibitor was severely corroded after 24 h immersion. The release of Ce(NO_3_)_3_ was able to mitigate the corrosion on the scratched area, resulting in a reduced amount of observed corrosion products. Both specimens with loaded NaVO_3_ and Na_3_PO_4_ could significantly suppress the corrosion, although localized corrosion sites could be observed at high magnification for the NaVO_3_-loaded specimen, as shown in Figure 24d–g. The considerably higher impedance of the scratched specimen loaded with Na_3_PO_4_ also confirm its superior self-healing properties (Figure 24h). 

The capillary effect can help the inhibitor solution to enter the micro/nano-size pores in PEO coatings. However, since the pores are semi-closed, the air trapped inside them often hinders the solution uptake [257,258]. Therefore, the effective way to load the pores with more inhibitor solution is to force out the air from the pores by applying low pressure during the loading [256]. Prolonging immersion time and increasing the number of successive immersions can increase the amount of inhibitor loaded inside the pores. However, excessive exposure of PEO film to the aqueous solution has been reported to have an adverse effect on the corrosion protection of the PEO coating [159,256,259,260]. The quantification of incorporated inhibitors in the PEO pores and their release rate in a corrosive medium is a determining factor for evaluating the ability and time of self-healing functionality. As an example, Liu et al. [256] incorporated Na_3_PO_4_ in a PEO coating through immersion in 0.5 M Na_3_PO_4_ for 10 min in a low-pressure vacuum condition. The PEO pores were then sealed with top paint. The concentration of the released phosphate from a scratched specimen exposed to 3.5 wt.% NaCl solution was quantified according to national standard of GB/T 9727-2007 (for details, see [256]). They reported that the concentration of released phosphate reaches a stable value after 7 days, meaning the phosphate is almost fully released within 7 days. Similar investigations on the release rate of the inhibitors from any type of coatings, which is a piece of valuable information to evaluate the self-healing ability of the coating, are unfortunately scanty in the literature [208,261,262]. 

The concentration of incorporated organic inhibitors on the PEO layer can be enhanced using a secondary substance loaded into the PEO and facilitate the adsorption of the organic substances. In a recent work by Al Zoubi et al. [263], TiO_2_ nano particles were incorporated into a PEO layer on a Mg alloy during the PEO formation process. Diethyl-5-hydroxyisophthalate (DEIP) was loaded into a PEO inorganic coating by immersion in 1 M ethanolic solution of DEIP. The presence of TiO_2_ particles resulted in a significantly higher amount of DEIP-contained layer on the PEO layer. As a result, the corrosion rate of the coated Mg was considerably improved, although the active corrosion inhibition was not discussed. 

**Figure 25:**
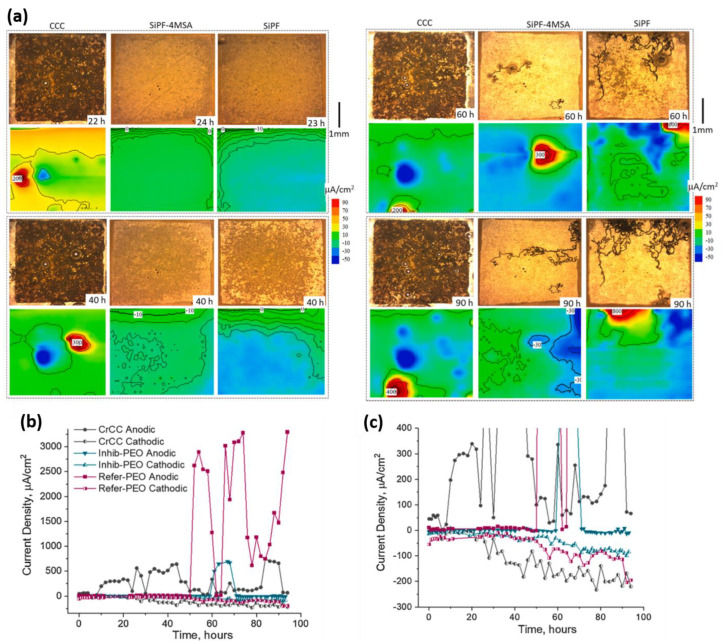
(**a**) Optical micropgraphs and SVET current density maps of reference CCC and PEO coatings with and without 4MSA inhibitor, acquired during immersion in 0.05 M NaCl. The immersion time is specified in each section of this Figure. (**b**,**c**) Evolution of the peak current density, anodic and cathodic, for three types of tested samples during 96 h of immersion in 0.05 M NaCl. “SiPF” refers to the reference PEO sample. Adapted [264] with permission from Elsevier.

In a recent work by Wierzbicka et al. [264], a PEO coating was formed on an AZ31 alloy in a solution containing silicate Na_2_SiO_3_, Na_3_PO_4_, and KF. Sodium salt of 4-Metylsalicylate (4MSA) was incorporated into the PEO pores via immersion in low-pressure condition. SVET maps over the sample surface (shown in Figure 25) revealed a rather unique corrosion suppression behavior. Filiform corrosion appeared on the samples with and without the inhibitor after approximately 60 h of immersion in 0.05 M NaCl solution, which was also reflected in the local cathodic and anodic activity in their SVET maps. The filiform corrosion on the sample with inhibitor was temporary and largely suppressed over time. Further immersion time revealed similar activation/suppression behavior at another location, demonstrating the endowed active corrosion protection with the incorporated 4MSA inhibitor. In contrast, the inhibitor-free sample exhibited a much higher local anodic current. Interestingly, in comparison to a commercial chromate conversion coating (CCC), the 4MSA-loaded PEO exhibited a lower corrosion activity within 96 h of immersion. Another isomer of the same inhibitor (3-Methylsalicylate) has also shown a strong active inhibition characteristic when it is loaded into a PEO coating on CP-Mg [262]. Some other recently discovered organic corrosion inhibitors, including sodium salts of glycolic, 4-aminosalicylic, and 2,6-pyridinedicarboxylic acids have been loaded into a PEO coating on AZ91 alloy and sealed with a thin sol–gel to prevent uncontrolled leaching [265]. The active corrosion protection of such composite coating was proven by continuous SVET measurements for 48 h.

Inhibitors can also be loaded into nanocontainers, such as halloysite nanotubes [60,266,267,268] and LDH, and subsequently applied onto PEO coating via immersion post-treatment. Such an approach yields more control on the release of inhibitors, which is triggered by the effect of corrosion reaction byproducts (e.g., increase in OH^−^ or Mg^2+^) or corrosive ions (e.g., Cl^−^ or SO_4_^2−^). Thus, active and long-term corrosion protection can be achieved [60]. Recent research has highlighted LDH’s potential to serve as nanocontainers in the PEO layer [30,254,260,261,269,270,271,272]. A LDH layer can be directly grown on the PEO layer, which also blocks the PEO pores and improves its barrier properties. 

**Figure 26:**
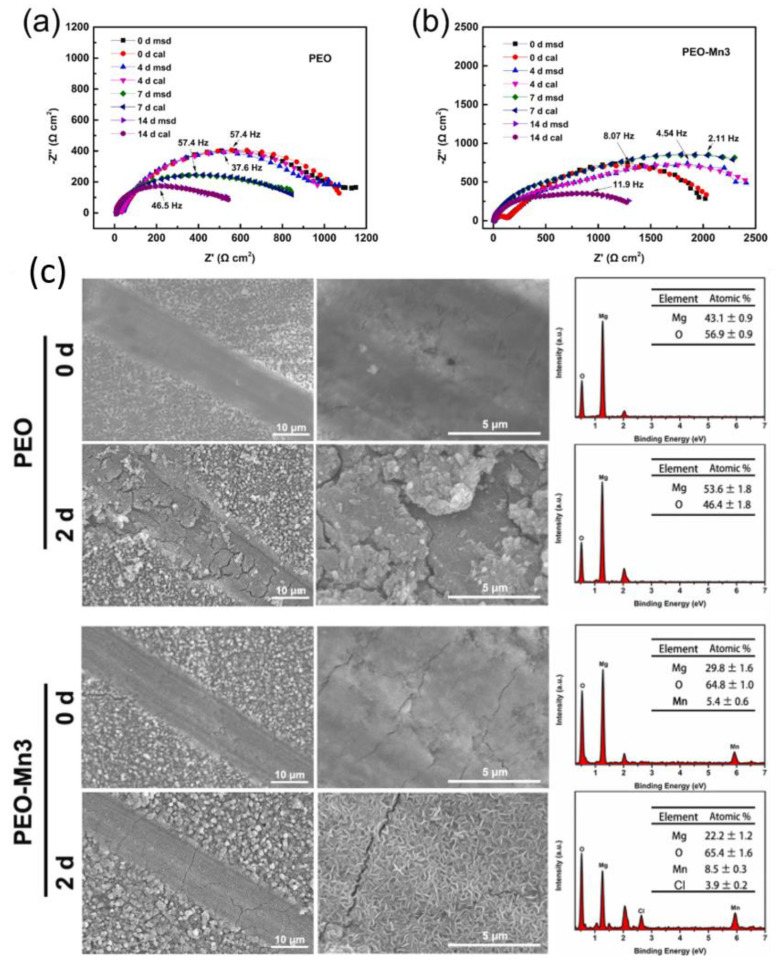
(**a**) Nyquist plots of the PEO sample without (**a**) and with (**b**) deposited MnOOH layer immersed in 0.9 wt.% NaCl solution for various times. (**c**) SEM surface views and EDS spectra of scratches area on the PEO sample without and with deposited MnOOH layer immersed in 0.9 wt.% NaCl solution for various times. PEO-Mn3 denotes the PEO-coated sample immersed in 12 g/L MnCl_2_ for 9 h. Adapted [273] with permission from Elsevier.

Recently, Zhang et al. [273] showed that the application of a thin MnOOH layer on a PEO coating via a simple immersion in MnCl_2_ solution can promote the formation of LDH flakes, which are supplied by the corrosion products of Mg dissolution (Mg^2+^ and OH^−^ ions). Active corrosion protection was confirmed in different ways, including the increase in the impedance value of the specimen during the immersion in a NaCl solution (Figure 26a,b), observing the LDH growth on an artificially defected zone (Figure 26c), and detecting the Cl^−^ on the defected zone (Figure 26c). A similar concept of promoting the formation of LDH film by inhibitors (in this case, Li) and as the response to corrosion of aluminum alloy was firstly proposed by Visser et al. [274,275,276,277,278,279].

**Figure 27:**
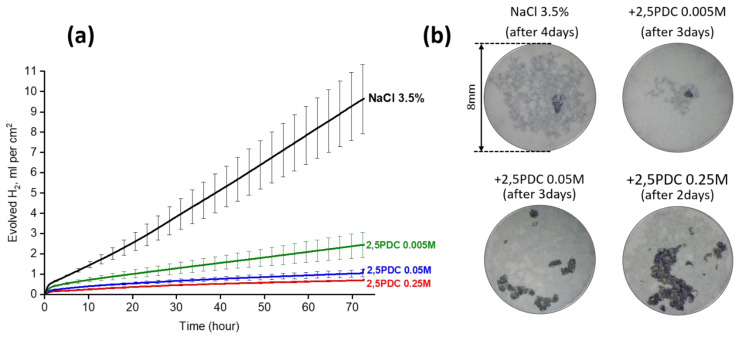
(**a**) H_2_ evolved during immersion of the AZ21 sample in 3.5 wt.% NaCl solution containing different concentrations of 2,5PDC. (**b**) The surface appearance of PEO-coated AZ21 after the failure (duration is specified in each case) in 3.5 wt.% NaCl solution containing different concentration of 2,5PDC. Adapted [159] with permission from Elsevier.

PEO coating is a ceramic film that usually features inertness toward many chemicals, including neutral inhibitor-containing aqueous solution. However, in a recent study, Vaghefinazari et al. [159] demonstrated that although the significant corrosion inhibition effect of 2,5PDC has been reported for several Mg alloys [10], it can deteriorate the protective properties of a PEO coating on Mg, which in turn, leads to overall faster degradation of PEO-coated Mg substrate. Figure 27a illustrates the reduction in the H_2_ evolution rate of bare AZ21 Mg with the increase in the concentration of 2,5PDCA in corrosive 3.5 wt.% NaCl solution. Meanwhile, in Figure 27b, the severe degradation of the same Mg substrate coated with PEO immersed in solutions with an increasing concentration of 2,5PDCA is shown. The adverse effect of 2,5PDC on the PEO coating is attributed to the formation of soluble complexes between 2,5PDC and Mg, which destabilize the PEO components. Moreover, the inhibition mechanism of 2,5PDC on bare Mg alloys has been shown to be tied to the formation of a relatively thick Mg(OH)_2_ film on the substrate [159]. The formation of such Mg(OH)_2_ layer in the PEO/Mg interface exerts mechanical force on the PEO layer atop, which in turn facilitates its detachment. Therefore, it is crucial to preliminarily investigate the interaction of an effective corrosion inhibitor with both a bare and PEO layer to foresee its potential adverse effects on the overall corrosion protection performance of the system. Such adverse effects of 2,5PDC have also been reflected in another recent report [260].

Zhang et al. [260] loaded a PEO layer on AZ31 with 0.1 M concentration of 2,5PDC and two other inhibitors, namely metavanadate and 5-aminosalicylate. The adverse effect of inhibitor impregnation on the initial (within 20 min) impedance of the samples immersed in 3.5 wt.% was observed. However, further immersion led to a slightly higher impedance value for the corresponding samples with metavanadate and 5-aminosalicylate inhibitors. To prevent the wasteful release of inhibitors into the electrolyte during immersion, the PEO pores were sealed via immersion in Ce(NO_3_)_3_ and oxidative H_2_O_2_ solution, which resulted in a CeO_2_ sealing layer. Eventually, MgAl-LDH was grown on PEO in an autoclave at 398° K. The composite coatings loaded with 5-aminosalicylate and sealed with CeO_2_ and LDH layers excelled in high protective properties, demonstrating 0.1–0.5 Gohm.cm^2^ low-frequency impedance during the first day of immersion in 3.5% NaCl. The results showed that the incorporated inhibitors can promote the formation of the LDH layer via modification of the Mg ion supply through complexation with Mg ions, possibly from both the PEO layer and Mg substrate. Nevertheless, a very complex active corrosion mechanism could be hypothesized from the loaded inhibitors, the cerium-containing layer, and the LDH layer.

The growing number of studies on PEO coating with incorporated corrosion inhibitors indicates the highly promising properties of PEO layer in storing the inhibitors. Combined use of protective strategies, i.e., loading of porous PEO layers with corrosion inhibitors alone or in nanocontainers, followed by sealing with hybrid or organic coatings loaded with inhibitor, provides enhanced long-term corrosion protection for magnesium alloys possessing strong barrier and active corrosion protection in case the coating is damaged. 

## 6. Summary and Perspectives

The acute needs of Mg corrosion protection research have been discussed, emphasizing the development of durable barrier corrosion protection, the continuing quest for replacement of carcinogenic chromates, and especially the ways of merging barrier and active protection by developing new corrosion-inhibiting coating technologies. Despite recent achievements uncovering new effective corrosion protection systems on Mg alloy, the quest continues to replace carcinogenic Cr(VI) from everyday corrosion protection practice. Durable barrier corrosion protection can be endowed with active corrosion inhibition by accommodating corrosion inhibitors, achieving the benchmark “self-healing” properties of Cr(VI)-based protection technologies. 

Despite the remarkable corrosion inhibition of several inorganic inhibitors, including vanadate, arsenate, and selenite, they are not likely to become industrially acceptable candidates for the replacement of Cr(VI) due to their environmental and toxicity issues. Nitrates and molybdates, given their seemingly universal and significant inhibition effects, have not received the dedicated attention they deserve. 

Given the vast number of organic molecules with potentially corrosion-inhibiting properties, computational (*in silico*) screening of the most effective corrosion inhibitors is the only way to find optimized solutions within a relatively short time. Machine learning, successfully applied to solve other chemical problems, allows rational design of bespoke inhibitors for each specific magnesium alloy. This will be much more time and cost efficient compared to a purely experimental approach. Representative and reliable experimental databases are needed for setting up the initial training space. A systematic approach to comparing the inhibiting efficiency of multiple corrosion inhibitors to specific alloys is needed, given the significant influence of individual alloy impurities and surface treatment. This calls for application of existing and development of new, reliable high-throughput testing techniques, potentially involving robotic testing. Collection of big datasets, however, does not substitute the in-depth study of inhibiting mechanisms of selected most effective or representative inhibitors.

Development of new experimental techniques and data processing methods is needed to alleviate the influence or benefit from critical factors related to testing conditions (elemental composition and microstructure of alloy, the composition and concentration of testing electrolyte, inhibitor concentration, initial pH, the ratio of exposed surface area to electrolyte volume, and eventually the testing time).

Loading coating systems with inhibitors is an effective approach to improve the overall corrosion protection performance and achieve active corrosion inhibition. PEO coatings feature porous microstructure to accommodate a high concentration of inhibitors, LDH coatings possess a more controlled release of the inhibitor and trap the aggressive chloride in exchange, and sol–gel coatings with potentially highly achievable barrier properties also can benefit from the loaded inhibitors, as long as their barrier properties are not impaired by the inhibitors. Unfortunately, despite the numerous combinations of coatings and inhibitor with remarkable corrosion protection performance, the “active” inhibition properties are commonly taken for granted and left without putting into examination, presuming a successful corrosion inhibition of bare Mg by the inhibitor. Thorough experimental validation of self-healing or active protection effect needs to be acquired, including that by (local) electrochemical methods and industrially relevant salt-spray tests or long atmospheric exposure.

In addition to the high corrosion protective performance of the reviewed inhibitor-loaded systems, their environmentally friendliness renders them as a suitable replacement technology to Cr(VI)-based coatings. Indeed, cost effectiveness and feasibility of developed inhibitor-loaded systems for engineering parts in industrial scale must be justified by the industry.

## Data Availability

Not applicable.
